# Immunization of pigs with replication-incompetent adenovirus-vectored African swine fever virus multi-antigens induced humoral immune responses but no protection following contact challenge

**DOI:** 10.3389/fvets.2023.1208275

**Published:** 2023-06-19

**Authors:** Michelle D. Zajac, Jessie D. Trujillo, Jianxiu Yao, Rakshith Kumar, Neha Sangewar, Shehnaz Lokhandwala, Huldah Sang, Kylynn Mallen, Jayden McCall, Leeanna Burton, Deepak Kumar, Emily Heitmann, Tristan Burnum, Suryakant D. Waghela, Kelli Almes, Juergen Richt, Tae Kim, Waithaka Mwangi

**Affiliations:** ^1^Department of Diagnostic Medicine/Pathobiology, Kansas State University, Manhattan, KS, United States; ^2^Department of Veterinary Pathobiology, Texas A&M University, College Station, TX, United States

**Keywords:** African swine fever (ASFV), polyvalent Ad5 vectored vaccine, natural transmission model, histopathology, real-time quantitative polymerase chain reaction (RT-qPCR), IgG

## Abstract

**Introduction:**

African swine fever virus (ASFV) is a pathogen of great economic importance given that continues to threaten the pork industry worldwide, but there is no safe vaccine or treatment available. Development of a vaccine is feasible as immunization of pigs with some live attenuated ASFV vaccine candidates can confer protection, but safety concerns and virus scalability are challenges that must to be addressed. Identification of protective ASFV antigens is needed to inform the development of efficacious subunit vaccines.

**Methods:**

In this study, replication-incompetent adenovirus-vectored multicistronic ASFV antigen expression constructs that covered nearly 100% of the ASFV proteome were generated and validated using ASFV convalescent serum. Swine were immunized with a cocktail of the expression constructs, designated Ad5-ASFV, alone or formulated with either Montanide ISA-201™ (ASFV-ISA-201) or BioMize^®^ adjuvant (ASFV-BioMize).

**Results:**

These constructs primed strong B cell responses as judged by anti-pp62-specific IgG responses. Notably, the Ad5-ASFV and the Ad5-ASFV ISA-201, but not the Ad5-ASFV BioMize^®^, immunogens primed significantly (*p* < 0.0001) higher anti-pp62-specific IgG responses compared with Ad5-Luciferase formulated with Montanide ISA-201™ adjuvant (Luc-ISA-201). The anti-pp62-specific IgG responses underwent significant (*p* < 0.0001) recall in all the vaccinees after boosting and the induced antibodies strongly recognized ASFV (Georgia 2007/1)-infected primary swine cells. However, following challenge by contact spreaders, only one pig nearly immunized with the Ad5-ASFV cocktail survived. The survivor had no typical clinical symptoms, but had viral loads and lesions consistent with chronic ASF.

**Discussion:**

Besides the limited sample size used, the outcome suggests that *in vivo* antigen expression, but not the antigen content, might be the limitation of this immunization approach as the replication-incompetent adenovirus does not amplify *in vivo* to effectively prime and expand protective immunity or directly mimic the gene transcription mechanisms of attenuated ASFV. Addressing the *in vivo* antigen delivery limitations may yield promising outcomes.

## 1. Introduction

African swine fever (ASF) is a virulent disease in domestic swine and wild boar that is caused by the African swine fever virus (ASFV) (1). The ASFV is a complex enveloped DNA virus in the family Asfarviridae (2). Epidemics caused by the ASFV have an overwhelming negative economic impact on the affected regions and jeopardize swine commerce globally with nearly 100% mortality in naïve populations (3, 4). The global spread of ASFV (Georgia 2007/1) has occurred rapidly since its introduction from Africa and into the Russian Federation in the same year (5). Since then, the spread has occurred in several countries including Belgium (2018), the People's Republic of China (2018), the Dominican Republic, and Haiti (2021) and has recently spread to Italy (2022) with additional reports in Northern Macedonia and Thailand (3, 6).

Eradication of ASFV is not currently achievable given its presence in domestic and wild suids in many countries including Sub-Saharan Africa where it is also present in ticks (1). Management of this pathogen is a much more feasible option. Control of ASFV dissemination has traditionally been through stomping out practices and implementing biosafety and security measures (7). Despite encouraging outcomes from studies that have evaluated vaccine candidates, including attenuated and inactivated ASFV, there are still safety concerns, such as the possibility of reversion, and poor efficacy (8–13). These vaccine development approaches need to overcome multiple challenges including poor induction of protective immunity, shedding of vaccine virus, increased post-vaccination reactions, and unpredictability in the effects these viral modifications will have (14).

Subunit ASFV vaccines have historically included limited antigens with varied success, ranging from a complete lack of protection to full protection in a limited number of immunized animals (15–17). Antigen delivery platforms, such as DNA vaccines and recombinant proteins or a combination of the two administered with or without adjuvant, have been evaluated (18–20). These approaches can induce robust immune responses, but variable protective efficacy has been reported (18–20). More recent innovations incorporate viral vectors for antigen delivery that stimulate strong and specific cellular immune responses, and in some instances, they have been shown to confer partial protection from ASF (21–24). Replication-deficient adenovirus type 5 (Ad5) and recombinant *Vaccinia virus* (rVACV) have recently been demonstrated as the most promising vector platforms for ASFV vaccination (23, 24). However, challenges remain such as (1) determination of correlates of immune protection; (2) identification of protective antigens; and (3) development of an efficacious formulation and determination of the most appropriate immunization route.

Development of an efficacious ASFV subunit vaccine has not been successful due, in part, to the large viral genome, which encodes more than 150 proteins and the protective antigens are yet to be identified (25). While numerous immunodominant ASFV antigens have been characterized to date, empirical determination as to which antigen is required for protection has yet to be resolved (18, 21–23, 25–27). Some experimental vaccines utilizing this approach were able to achieve delayed viremia and death (and in some cases limited protection from disease) using one to multiple ASFV antigens (17, 23, 28). Recombinant *Vaccinia virus* encoding a combination of ASFV antigens (formulated without adjuvant) generated promising results with reduced blood and tissue viremia, even though protection from infection was not achieved following challenge (24). Available data suggest that the design of a viral vectored vaccine for ASFV is expected to require the inclusion of multiple protective antigens. The major drawback to this design is in the time needed to (1) empirically determine which antigen(s) to be included; (2) generate the expression constructs; and (3) test and determine the most efficacious formulation in domestic swine. Despite these limitations, the use of defined ASFV antigens for vaccination may offer a safer immunization option and has shown great potential in stimulating antigen-specific immune responses when packaged in a viral vector (23, 24, 29, 30). Delivery of multiple antigens can be achieved by use of multicistronic expression cassettes that utilize the 2A cleavage motif to allow the generation of multiple independent antigens from a single mRNA molecule (31–33).

In the current study, safety, immunogenicity, and protective efficacy of a replication-incompetent Ad5-vectored prototype subunit vaccine encoding ASFV multicistronic expression cassettes were evaluated in piglets. The experimental vaccines were formulated with no adjuvant or with either Montanide ISA-201™ or BioMize^®^ adjuvant and used to immunize domestic piglets in a homologous prime-boost strategy. Vaccine efficacy was evaluated using a natural ASFV transmission model by exposure to comingled naïve ASFV-infected spreaders (24, 28, 34–36).

## 2. Materials and methods

### 2.1. Generation of recombinant plasmid and adenovirus constructs

Selected ASFV Georgia 2007/1 open-reading-frames (Gene bank Accession FR682468) were used to design and generate multicistronic expression cassettes (Table 1). The pp220 polypeptide was split into two due to its large size. The polypeptide sequences were used to generate codon-optimized synthetic genes (GenScript, NJ, USA) that were cloned into pcDNA3.1+ vector (Invitrogen, K8300001, CA, USA) with N-terminal HA and C-terminal FLAG tags, respectively. Protein expression by the recombinant plasmid constructs was evaluated by immunocytometric analyses as previously described (21–23, 37). The outcome was used to select the best expressers that were used as templates to PCR amplify the genes, which were then used to assemble cognate recombinant replication-incompetent adenoviruses using the Invitrogen ViraPower Adenoviral Expression System (K493000, CA, USA) as previously described (21–23, 37). A recombinant replication-incompetent adenovirus expressing luciferase (Ad5-Luciferase) was similarly generated. The recombinant adenoviruses were scaled up, and viral titers (IFU/mL) were determined by immunocytometric analyses as previously described (21, 22).

**Table 1:** Ad5-ASFV constructs.

**Construct**	**ASFV antigens**	**Construct**	**ASFV antigens**
Ad5-01	CP2475 (p220) (1-1256aa)	Ad5-22	F1055L, E146L, I8L
Ad5-02	CP2475 (p220) (1190-2476aa)	Ad5-23	B962L, H233R, E75, H171R
Ad5-03	p72, p15, B602L,	Ad5-24	C962R, MGF505-3R, C147L
Ad5-04	pp62, p32, p54, EP153R, p10	Ad5-25	A859L, B318L, B169L
Ad5-05	K205R, A104R, EP402R, A151R, B119L, K196R, BA71V-CP80R	Ad5-26	C717R, H359L, F317L
Ad5-06	B438L, R298L, NP419L, K145R	Ad5-27	MGF505-10R, B475L, MGF360-4L
Ad5-07	B385R, CP312R, F165R	Ad5-28	MGF505-2R, B407L, MGF360-13L, E111R
Ad5-08	F778R, S273R	Ad5-29	K421R, EP424R, D339L, S183L
Ad5-09	NP868R, H339R	Ad5-30	EP296R, B263R, C257L, I243L, A179L, B117L
Ad5-10	I329L, A224L, MGF505-6R	Ad5-31	Q706L, D205R, E184L, I177L, C84L, L60L, DP60R
Ad5-11	C475L, B354L, D345L, H124R	Ad5-32	MGF505-10R, MGF300-1L, E199L, DP96R, EP84R, DP79L, DP71L, X69R
Ad5-12	C315R, MGF505-7R, MGF300-1L	Ad5-33	MGF505-11L, I267L, I196L, C129R, MGF100-1R, MGF110-3L
Ad5-13	MGF360-6L, MGF360-12L, MGF300-4L, D205R	Ad5-34	MGF505-7R, E301R, MGF110-1L, I215L, O174L
Ad5-14	MGF360-4L, MGF360-15R, A238L, H240R, B125R	Ad5-35	QP509L, QP383R, MGF360-16R, I226R
Ad5-15	NP1450L	Ad5-36	MGF505-4R, MGF360-9L, MGF360-8L, A240L
Ad5-16	G1340L	Ad5-37	MGF505-9R, EP364R, E248R, A137R, D129L
Ad5-17	M1249L, A118R, 173R	Ad5-38	MGF505-5R-MGF360-3L-MGF360-18R-DP238L-DP63R
Ad5-18	EP1242L, I9R, C62L,	Ad5-39	M448R-E423R-MGF505-1R
Ad5-19	G1211R, I7L, L83L,	Ad5-40	MGF505-6R-CP123L-I10L-I8L-MGF110-12L-I9R-L11L-MGF110-7L- MGF110-2L
Ad5-20	P1192R, EP152R, E66L,	Ad5-41	MGF360-4L-MGF360-2L-MGF360-11L-MGF110-9L
Ad5-21	D1133L, E165R, C122R	Ad5-42	MGF505-5R, DP238L

### 2.2. Validation of protein expression by the recombinant Ad5 virus constructs

**Figure 1:**
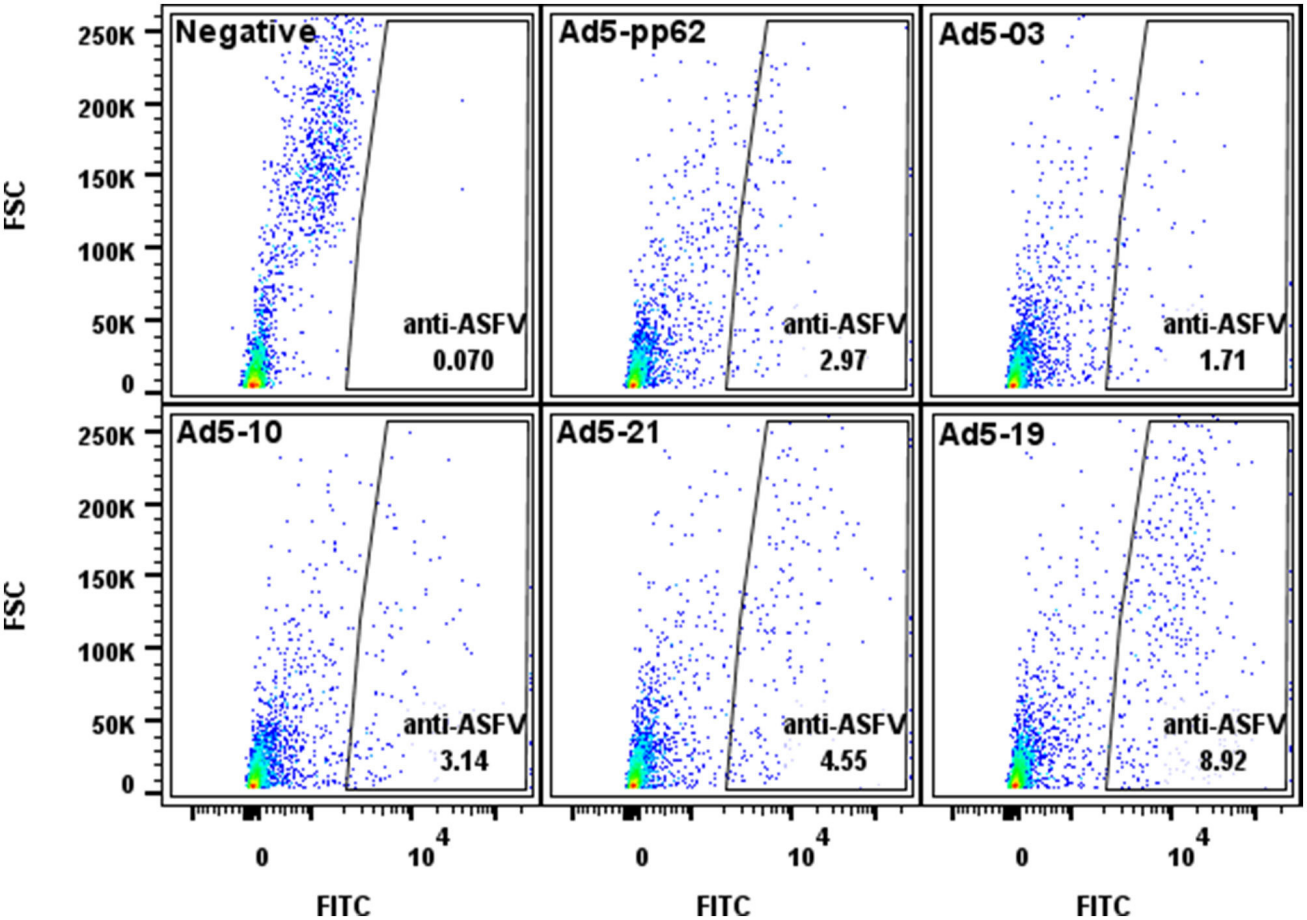
Flow cytometric analyses of protein expression by the Ad5-constructs. Dot plot presentation of forward scatter (FSC) vs. FITC for HEK293A cells infected with representative low (Ad5-03), medium (Ad5-10 and Ad5-21), and high (Ad5-19) antigen expressing Ad5-ASFV constructs followed by staining using ASFV convalescent serum. Ad5-pp62 served as the positive control and non-infected cells served as the negative control. Protein expression by representative Ad5-ASFV constructs is presented in Supplementary Table 1.

Flow cytometry was used to evaluate protein expression by each recombinant adenovirus construct. Briefly, human embryonic kidney (HEK) 293A cells were infected with Ad5-ASFV constructs and harvested after 24 h using 2 mM EDTA to lift cells from the bottom of the culture vessel. Cells were then distributed at 1 × 10^6^ cells per 5-mL polystyrene snap cap tube for each construct, followed by washing 2X using the Cyto-Fast™ Fix/Perm Buffer Set (BioLegend, 426803, CA, USA). Briefly, following the manufacturer's instructions, 1 mL wash buffer was added to each tube, gently vortexed, and centrifuged at 900 rpm for 5 min, after which the supernatant was discarded. Duplicate tubes were probed with a 1:200 dilution of ASFV-specific convalescent swine serum (26–28, 38) for 20 min at 4°C (kept in the dark). After washing as above, the cells were then probed with goat anti-porcine IgG FITC secondary antibody (Jackson ImmunoResearch, 114-095-003, PA, USA) diluted 1:250 in wash buffer for 20 min in the dark at 4°C. The cells were washed and fixed as per the manufacturer's instructions and resuspended in 2% goat sera (prepared in 1X PBS) before data acquisition using the BD LSRFortessa™ flow cytometer and BD FACSDiva™ followed by analyses using FlowJo software (BD Biosciences, OR, USA) (Figure 1). Negative mock-infected cells (media alone) were included and probed with ASFV convalescent serum and secondary antibody as controls for infection and gating.

#### 2.2.1. Immunization of pigs

**Figure 2:**
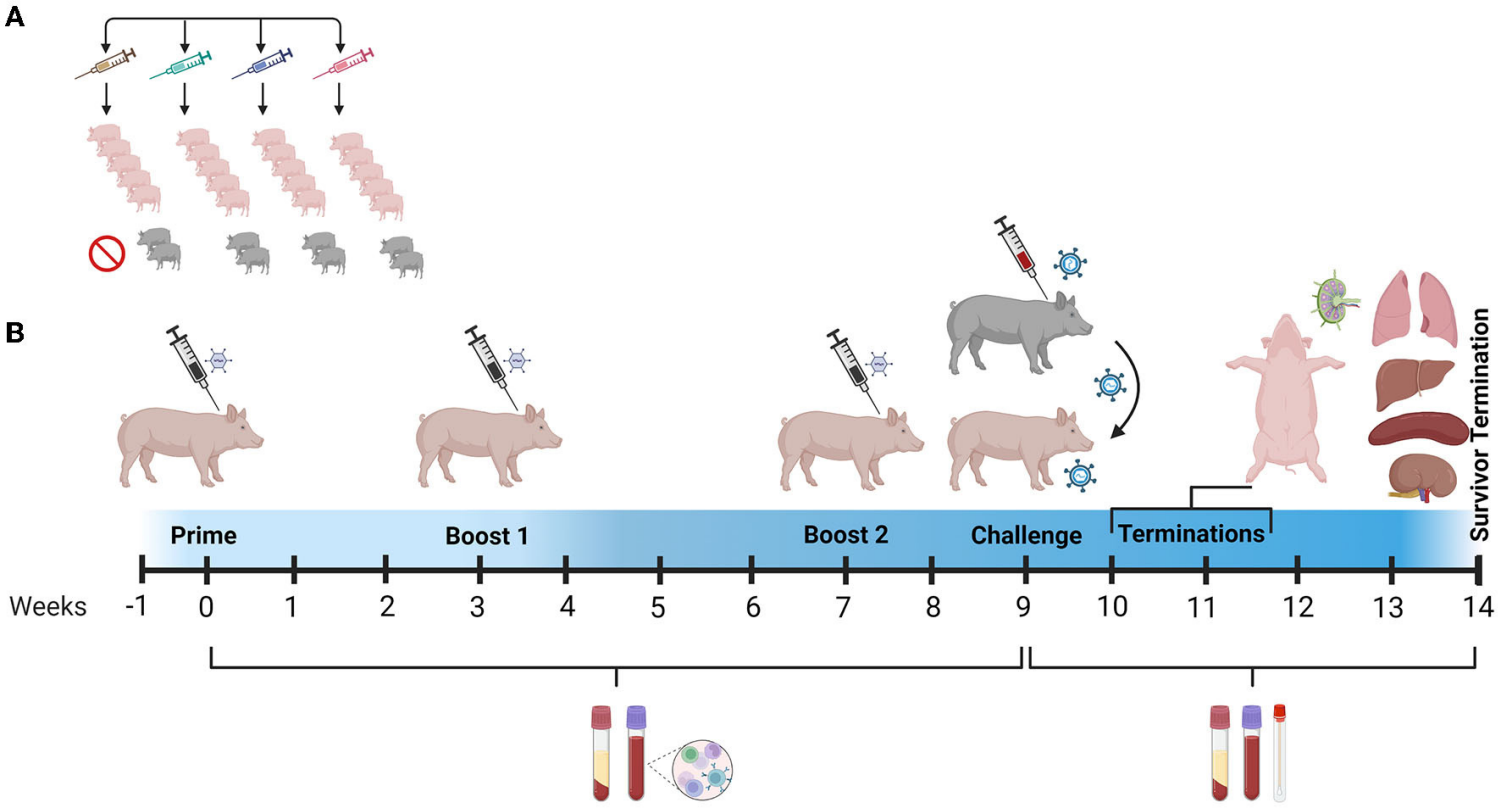
Study design and timeline. **(A)** Pigs were randomly assigned to four treatment groups: (1) Ad5-Luciferase plus Montanide ISA-201™ adjuvant (Luc-ISA-201); (2) Ad5-ASFV cocktail (ASFV-No Adjuvant); (3) Ad5-ASFV cocktail plus Montanide ISA-201™ adjuvant (ASFV-ISA-201); or (4) Ad5-ASFV cocktail plus BioMize^®^ adjuvant (ASFV-BioMize). **(B)** Animals were acclimatized for 1 week and baseline serum and PBMC samples were collected with weekly collections thereafter. Pigs in all the treatment groups were primed after acclimatization followed by boosting at weeks 3 and 7 post-priming as shown in Table 2. The pigs were challenged by contact with comingled ASFV-infected naïve pigs at week 9. Blood and nasal swabs were collected until termination. The survivor from the ASFV-No Adjuvant group was terminated 37 days post-challenge. Created with BioRender.com (accessed on 17 April 2023).

Twenty-eight piglets were acquired from a commercial vendor, housed at the BSL-2 Large Animal Research Center (LARC) at Kansas State University (KSU), and acclimatized for 1 week before immunization (Figure 2). The Ad5-ASFV cocktail (10^10^ ifu/construct, 4.2 × 10^11^ ifu total) was first diluted to the required concentration using phosphate-buffered saline (PBS) and then formulated with either no adjuvant, a 55:45 (w/w) ratio of Montanide ISA-201™ (Seppic, NJ, USA), or a 50:50 ratio of ready-to-use BioMize^®^ (VaxLiant, NE, USA) adjuvant and used to immunize pigs (*n* = 5) intramuscularly as shown in Figure 2, Table 2, and as previously described (23). Negative control pigs were similarly immunized, but with an equivalent dose of the Ad5-Luciferase (4.2 × 10^11^ ifu total) formulated with Montanide ISA-201™ adjuvant. Briefly, to form the Montanide ISA-201™ water-in-oil-in-water emulsion, the Ad5-ASFV cocktail or Ad5-Luciferase and adjuvant were first warmed to 37°C, followed by dropwise addition of the cocktail to adjuvant. Subsequently, each was mixed with a magnetic stir bar and plate set to low speed for 5 min at room temperature and incubated for an additional 30 min at room temperature without stirring. The inoculum was then transferred to 4°C for a final 1-h incubation, again without stirring. The pigs were boosted twice with the same priming dose and cognate formulation via the same route, 3 and 7 weeks post-priming. Each group had an additional two naïve pigs that were included to serve as contact spreaders in the challenge phase (Figure 2, Table 2).

**Table 2:** Immunization protocol.

**Groups**	**Pig ID**	**Dose per construct**	**Total dose per pig**	**Adjuvant**
CS^*^ Group 1	6908	none		
	6886			
Luc-ISA-201	6894	4.2 × 10^11^ ifu	4.2 × 10^11^ ifu	Montanide ISA-201™
	6909			
	6896			
	6897			
	6888			
CS^*^ Group 2	6915	None		
	6901			
ASFV-No adjuvant	6898	1 × 10^10^ ifu	4.2 × 10^11^ ifu	No adjuvant
	6903			
	6893			
	6892			
	6900			
CS^*^ Group 3	6887	none		
	6906			
ASFV-ISA-201	6884	1 × 10^10^ ifu	4.2 × 10^11^ ifu	Montanide ISA-201™
	6885			
	6883			
	6902			
	6910			
CS^*^ group 4	6912	none		
	6907			
ASFV-BioMize	6913	1 × 10^10^ ifu	4.2 × 10^11^ ifu	BioMize^®^
	6891			
	6890			
	6899			
	6904			

^*^CS, Contact spreaders. No inoculum or adjuvant received and direct intramuscular challenge.

#### 2.2.2. Sample collection and clinical scoring post-immunization

During the immunization phase, blood, body temperatures, and weights were collected before vaccination and weekly thereafter. Following immunization, vaccine safety and tolerability were determined by observing the pigs daily and the following parameters were monitored and recorded: body weight, injection site reaction, rectal temperature, coughing, nasal and ocular discharges, and signs of depression.

### 2.3. Evaluation of antibody responses

Immunogenicity of the Ad5-ASFV immunogens in pigs was determined by tracking pp62-specific IgG responses by ELISA as this antigen is highly immunogenic and sufficient amounts of mammalian cell-expressed recombinant protein can readily be generated (23). The ELISA was conducted using a pp62 concentration of 1 μg/mL (100 μL/well) to coat microplates and a 1:100 dilution of serum samples as previously described (21–23, 37). In brief, plates were first blocked with 5% non-fat dry milk (diluted in PBST: PBS + 0.1% Tween 20) followed by the addition of diluted serum (in triplicate) for 1 h at 37°C. After six repeated washes (using PBST), a 1:5000 dilution of anti-porcine IgG-POD (Jackson ImmunoResearch, 114-035-003, PA, USA) secondary antibody was added to each well and the plates incubated at 37°C for 1 h. The plates were washed 6× with PBST followed by 3× with PBS, and the POD colorimetric reaction was developed by adding Sure Blue tetramethylbenzidine (TMB) substrate (53-00-02, KPL, MA, USA). A 1N HCl solution was added to stop color development after 10 min, and the optical density (OD) was measured at 450 nm using a BioTek Epoch spectrophotometer (VT, USA). An irrelevant antigen, TMSP7, was used as a background control to establish the baseline for each sample which was subtracted from the pp62 OD value. Additionally, a previously validated ASFV convalescent serum was included in each plate as a positive control alongside a validated naïve swine serum.

### 2.4. Validation of Ad5-ASFV-induced antibodies

Recognition of wild-type ASFV by the antibodies induced by the Ad5-ASFV immunogens was determined by indirect fluorescence antibody (IFA) using ASFV (Georgia 2007/1)-infected and mock-infected naïve swine peripheral blood mononuclear cells (PBMCs) as previously described with the following modifications (21, 22). Briefly, PBMCs were thawed from liquid nitrogen, washed using cold complete RPMI (cRPMI) plated at a density of 1 × 10^6^ cells/well in a 96-well plate and incubated overnight at 37°C. The cells were infected using a MOI of 0.1 of ASFV (Georgia 2007/1) prepared in cRPMI and incubated at 37°C for 1 h after which infection media were discarded and replaced with fresh cRPMI. Non-infected cells were included to serve as negative controls. After 48 h, the cells were washed twice using PBS, fixed using ice-cold methanol (100%), and air-dried before blocking.

To perform the IFA, the plates were washed 2× using PBS and incubated with blocking buffer (2% bovine serum albumin (BSA) in PBS) for 1 h at 37°C. After blocking, the cells were probed with a 1:20 dilution of serum (from 1 week after the final boost) prepared in the blocking buffer for 1 h at 37°C. The ASFV-specific convalescent serum diluted at 1:500 was used as a positive control, and a 1:20 dilution of normal swine serum (Vector Laboratories, S-4000-20, CA, USA) was used as a negative control. Following three rinses with PBS, the wells were then incubated with 1:200 goat anti-swine IgG FITC (Jackson ImmunoResearch, 114-095-003, PA, USA) alongside additional wells probed with FITC only as controls for the secondary antibody for 45 min at 37°C. Two more washes were performed using PBS followed by the addition of 100 μL PBS to each well before microscopic examination. The cells were visualized, and images were acquired using an EVOS fluorescent imaging system (Thermo Fisher, MA, USA).

### 2.5. ASFV challenge

Two weeks after the final boost, the pigs were moved to the ABSL-3Ag biocontainment facility within the BRI on the KSU campus, acclimatized, and then challenged by exposure to contact spreaders infected with ASFV (Georgia 2007/1) (Figure 2). The two contact spreaders in each group were inoculated intramuscularly (IM) with a dose of 10^2^ TCID_50_/mL as determined by our previous study (23). The challenge of the comingled vaccinees in each group (*n* = 5) occurred via the natural transmission where infection occurs through direct contact with the infected spreaders or virus shedding in the pens which mimic the natural course of infection in field settings (23, 36).

### 2.6. Sample collection and clinical scoring post-challenge

Prior to initiation of challenge in the ABSL-3Ag biocontainment, baseline nasal swabs, whole blood, and blood for serum were collected. Following challenge, blood, nasal swabs, weights, and rectal temperatures were collected on days 0, 5, 7, 11, and 14 with clinical signs monitored twice daily using a scoring rubric (Supplementary Table 2). The surviving pig had additional collections on days 18, 21, 25, 28, 32, 35, and 37. Pigs that developed severe ASF were humanely euthanized followed by necropsy. The following tissues were collected: cranial mediastinal, gastrohepatic, mandibular, mesenteric, and renal lymph nodes; kidney, liver, lung, spleen, and tonsil.

### 2.7. Tissue pathology

Formalin-fixed tissues were processed for histological analysis using standard procedures (39). Histological lesions were evaluated and scored for all animals utilizing the standardized parameters established previously by Galindo-Cardiel et al. with adaptations as per Sunwoo et al. (20, 39). Systematic histological assessment was performed blindly on the spleen, tonsil, mandibular, cranial mediastinal, mesenteric, gastrohepatic, renal lymph nodes, lung, liver, and kidney. The scoring matrix was as follows: absent (0) or minimal (1), mild (2), moderate (3), or severe (4). Lesion categories scored included the following: tissue necrosis (such as infarction) and cellular necrosis (such as lymphocytolysis); fibrin thrombi, fibrin deposition in tissue or fibrinoid degeneration of vessels, congestion and/or hemorrhage, and inflammation such as infiltrates of macrophages, eosinophils, or neutrophils. Overall total organ and lymphoid organ score ranges are represented as minimal (0–30), mild (31–60), moderate (61–90), or severe (above 90). The total score ranges for viscera (lung, liver, and kidney tissues) are indicated as minimal (0–10), mild (11–20), moderate (21–30), or severe (above 30).

### 2.8. Evaluation of ASFV viremia

The detection and quantification of the ASFV genome in blood, swabs, and tissue samples from all pigs were performed using DNA purification followed by viral load assessment using a validated ASFV p72-specific quantitative real-time PCR (qPCR) assay. Briefly, ASFV DNA was extracted and purified from samples using the automated magnetic bead KingFisher Flex equipment (ThermoFisher, 5400610, MA, USA) that utilizes the Total DNA Extraction Kit (GeneReach, Taiwan) as previously described (20). Quantitative real-time PCR for the detection of the ASFV gene encoding the p72 antigen (primers: Integrated DNA Technology, IA, USA, and probe: ThermoFisher, MA, USA) was performed in duplicate wells using PerfeCTa^®^ FastMix^®^II (Quanta Biosciences, MA, USA) and following reagent and cycle parameters as previously described on the Stratagene Mx3005p Real-Time PCR Detection System (Agilent, CA, USA) (20, 40).

For the quantification of ASFV copy number (CN), serial dilutions (10×) of the positive control (ASFV p72 plasmid) were used to generate an eight-point standard curve (10^9^ to 10^1^ copies) using 16 qPCR well replicates performed as two scientific replicates. The ASFV p72 CN/reaction was mathematically determined using the mean cycle threshold value (Ct) and the slope and intercept of the DNA standard curve. A cycle threshold (Ct) cutoff of 38 for both PCR replicates was used as the positive cutoff. Each extraction and qPCR run included a standardized ASFV sample for the extraction and PCR-positive control as well as a negative control sample (molecular grade water) as an extraction negative and PCR no template control.

### 2.9. Statistical analysis

GraphPad Prism, version 9.5.0, was used to analyze the data as follows: The percent survival significance was determined by the Mantel–Cox test; antibody responses were assessed using the Ordinary one-way ANOVA followed by Tukey's multiple comparisons test, and virus titers and histological scores were analyzed using the two-way ANOVA followed by Tukey's multiple comparisons test. Each comparison evaluated the treatment groups against the mock-immunized negative control (Ad5-Luciferase) group as well as each immunization group against each other (ASFV-No adjuvant vs. ASFV-ISA-201 and ASFV-BioMize; ASFV-ISA-201 vs. ASFV-BioMize). For viremia and histological evaluation, all groups including the contact spreaders were compared for statistical significance. FlowJo, version 10.8.1, was used to gate and calculate the protein expression in the cells infected with the recombinant Ad5 virus constructs.

### 2.10. Ethics statement

Kansas State University Institutional Animal Care and Use Committee (IACUC) (Protocol # 3871 and #4411) and Institutional Biosafety Committee (IBC) (registration #1481) follows the regulations, policies, and guidelines put forth by the Animal Welfare Act, United States Department of Agriculture (USDA) Animal Care Resource Guide, and the Public Health Service (PHS) Policy on Humane Care and Use of Laboratory Animals. All protocols outlined in this document were followed including the use of clinical scoring for daily monitoring and assessment of animal health with weight and temperature included in this evaluation.

## 3. Results

### 3.1. Design, expression, and validation of ASFV antigen expression constructs

Recombinant pcDNA3 plasmid constructs encoding 42 ASFV expression cassettes, with HA and FLAG tags at the 5′ and 3′ ends, respectively, were generated (Table 1). The pp220 polyprotein was split into two constructs due to its large size, whereas one construct was generated for each one of the next two largest antigens, NP1450L and G1340L (Table 1). The pcDNA3 constructs expressed the encoded antigens as judged by immunocytometric analyses of transfected HEK293A cells probed with tag-specific mAbs or ASFV convalescent serum [expression data obtained as before (21, 22, 37)] with additional pp220 polyprotein expression previously demonstrated by Western Blot (37). The ASFV antigen expression cassettes from the pcDNA3 constructs were used to generate recombinant replication-incompetent adenoviruses (Table 1), and protein expression was confirmed by flow cytometric analyses of infected HEK293A cells probed with the ASFV convalescent serum (Figure 1). Protein expression by the recombinant adenoviruses was heterogeneous with low, medium, and high expression levels noted (Figure 1, Supplementary Table 1). Notable low expressers include Ad5-05 and Ad5-13, medium expressers include Ad5-04 and Ad5-29, whereas Ad5-09 and Ad5-19 were high expressers (Supplementary Table 1).

### 3.2. The Ad5-ASFV cocktail was well tolerated

**Figure 3:**
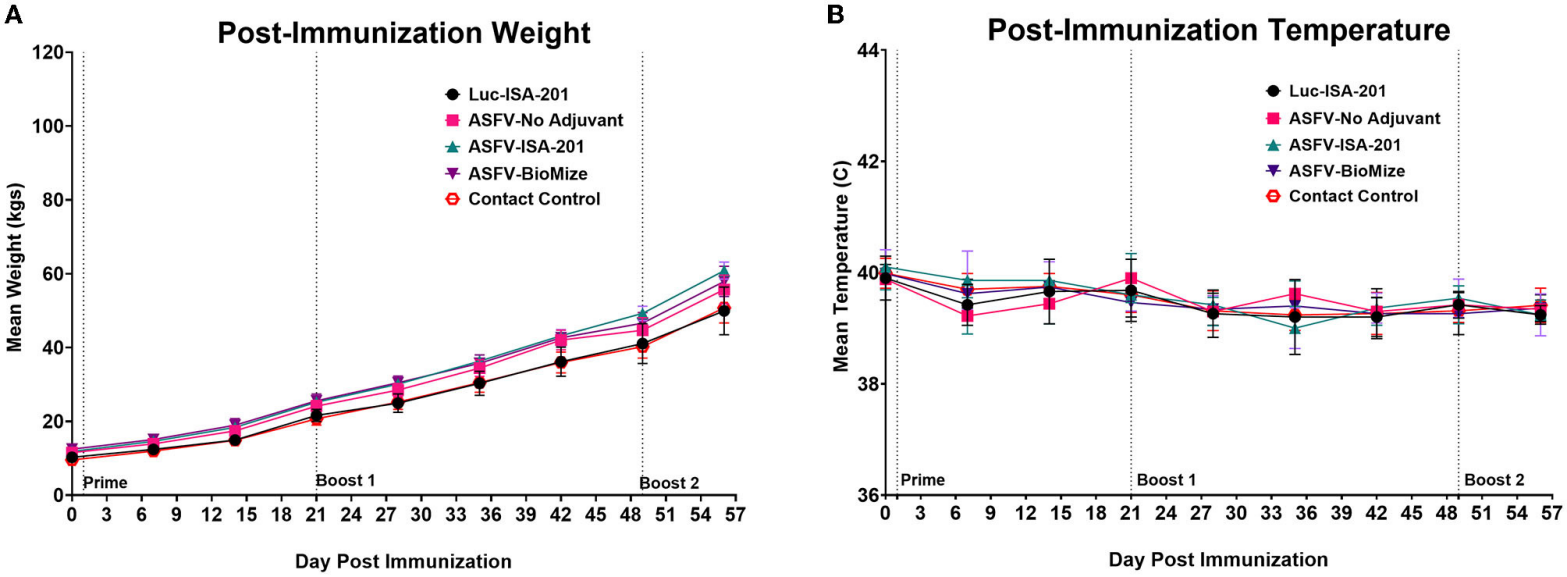
Post-immunization body temperatures and weights. **(A)** Weekly weight gain and **(B)** temperature change post-immunization. Average weekly weight gain and temperature change as clinical indicators of immunization effects for each group were plotted as group averages. Prime-boost immunizations are indicated on the days of administration. Contact Spreaders were not immunized and are indicated with a red open octagon.

Homologous prime-boost immunization of piglets with a cocktail of the recombinant adenoviruses expressing multiple ASFV antigens, designated Ad5-ASFV, alone or formulated in adjuvant (Figure 2, Table 2), was well tolerated as there was no adverse effect observed. Notably, the vaccinees, the negative controls, and the comingled non-immunized piglets that were included to serve as contact spreaders at the challenge stage were healthy, had normal weight gain, and maintained normal body temperatures throughout the immunization phase (Figure 3).

### 3.3. Ad5-ASFV cocktail induced strong antibody responses that recognized ASFV

**Figure 4:**
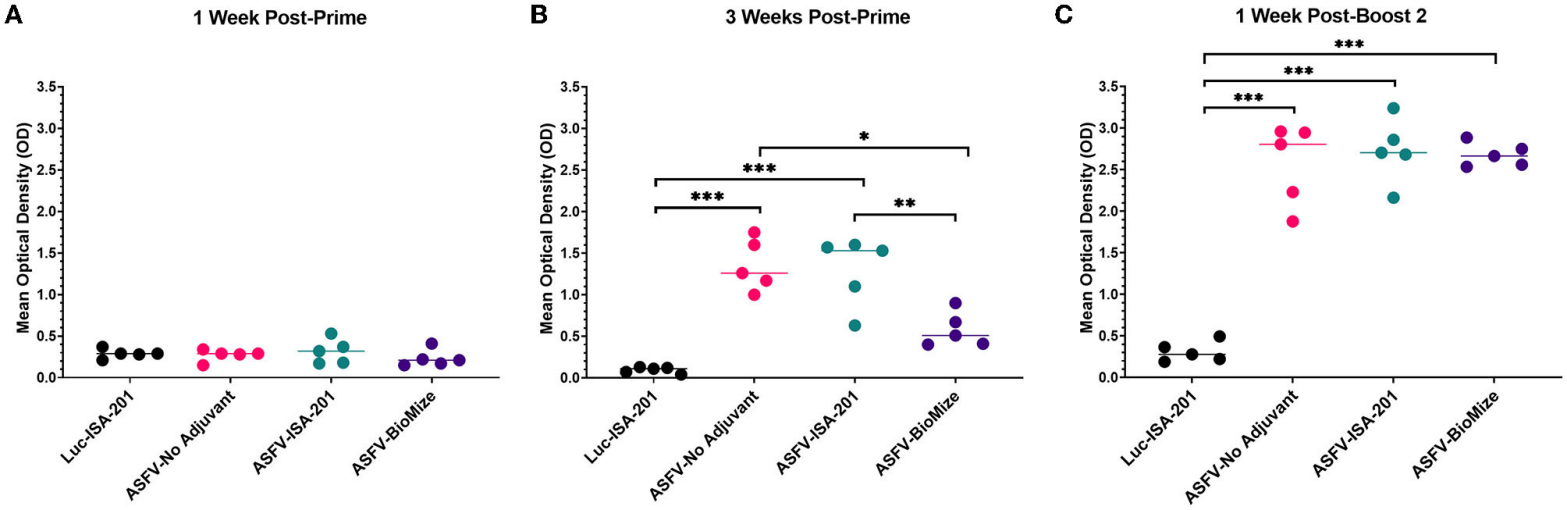
Ad5-ASFV elicited antibody responses. Following priming and boosting, recombinant pp62 antigen was used to track IgG responses by ELISA using sera from blood collected: **(A)** 1-week post-priming; **(B)** 3 weeks post-priming; or **(C)** 1 week after the second boost. Mean responses for the treatment groups are denoted by bars and statistically significant differences between the groups are denoted by asterisks, *p = 0.002, **p = 0.005, ***p < 0.0001, any groups not denoted with an asterisk were not statistically significant.

**Figure 5:**
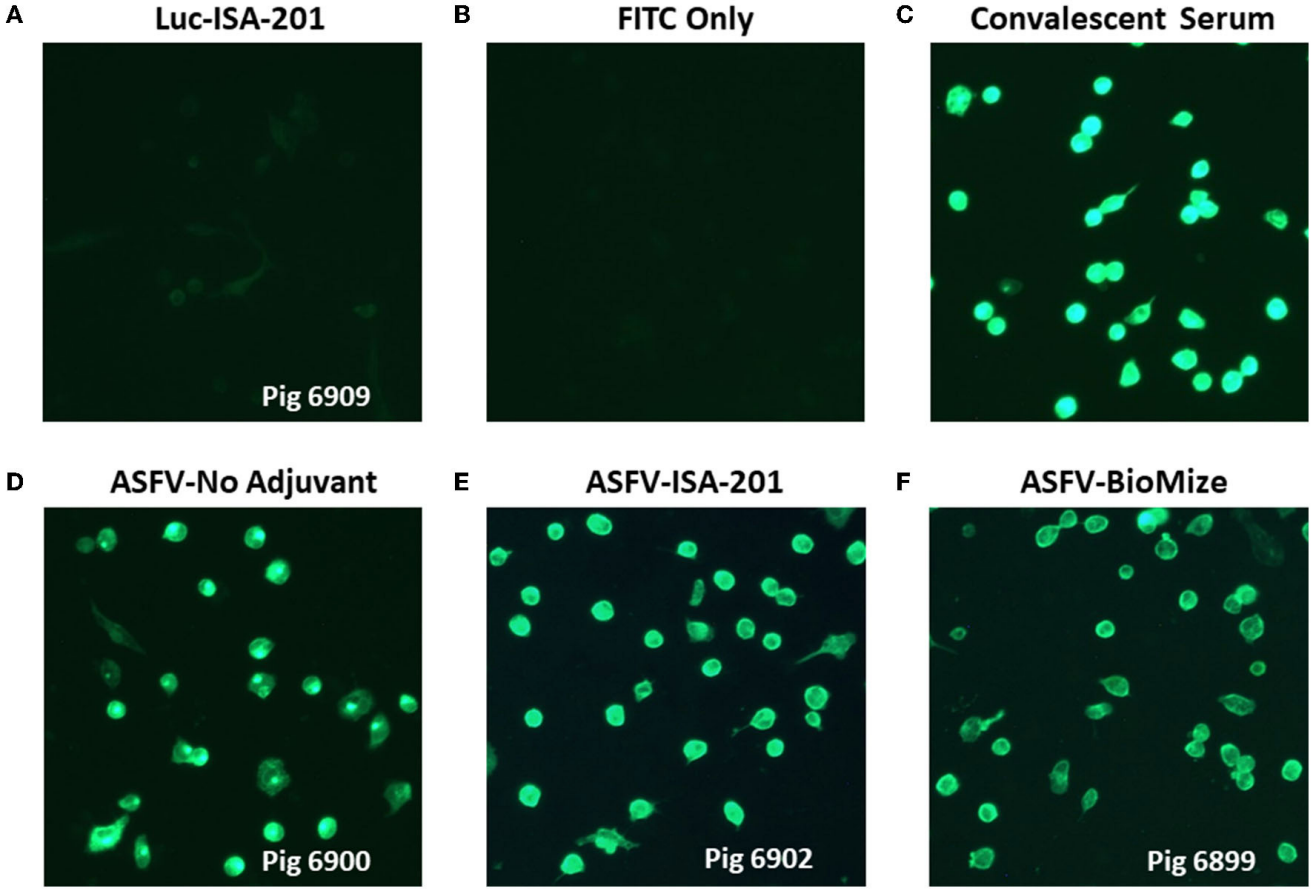
Antibodies elicited by Ad5-ASFV recognized ASFV-infected cells. Antibodies induced by the Ad5-ASFV formulations recognized ASFV-infected swine PBMCs as judged by IFA using sera from blood collected one-week after the second boost. Data for representative pigs from the negative control (6909) and the vaccines (6900, 6902, and 6889) is shown.

Immunization of piglets with the Ad5-ASFV cocktail, but not the negative control Ad5-Luciferase, primed ASFV-specific humoral immune responses in all the vaccinees as judged by pp62-specific IgG responses, which were used to evaluate immunogenicity (Figures 4A, B). Compared with the negative control Ad5-Luciferase Montanide ISA-201™ immunogen, the priming dose of the Ad5-ASFV cocktail alone or formulated with the Montanide ISA-201™ adjuvant elicited significantly (*p* < 0.0001) higher pp62-specific IgG responses, but the IgG responses primed by the Ad5-ASFV cocktail formulated in BioMize^®^ adjuvant was not significant (Figure 4B). The pp62-specific IgG responses primed by the Ad5-ASFV cocktail alone or the Ad5-ASFV cocktail formulated with the Montanide ISA-201™ adjuvant were significantly (*p* = 0.002 and *p* = 0.005, respectively) higher than the pp62-specific IgG responses induced by the Ad5-ASFV cocktail formulated in BioMize^®^ adjuvant (Figure 4B). The IgG responses primed by all the three formulations were significantly (*p* < 0.0001) amplified in all the vaccinees following the second booster dose (Figure 4C). Compared with the pp62-specific IgG responses induced by the priming dose, the second booster dose significantly (*p* < 0.0001) amplified the primary response (Figures 4B, C). However, following boosting, there was no significant difference in pp62-specific IgG responses between the pigs immunized with the Ad5-ASFV cocktail alone and the pigs immunized with the Ad5-ASFV cocktail formulated with either the Montanide ISA-201™ or the BioMize^®^ adjuvant, suggesting that these adjuvants did not have a booster effect on the pp62-specific IgG responses (Figure 4C). Immunization of piglets with the Ad5-ASFV cocktail, but not the Ad5-Luciferase immunogen, elicited antibodies that strongly recognized primary swine cells infected with wild-type ASFV (Georgia 2007/1) as judged by IFA using post-boost sera (Figure 5).

### 3.4. Ad5-ASFV cocktail did not confer protection

**Figure 6:**
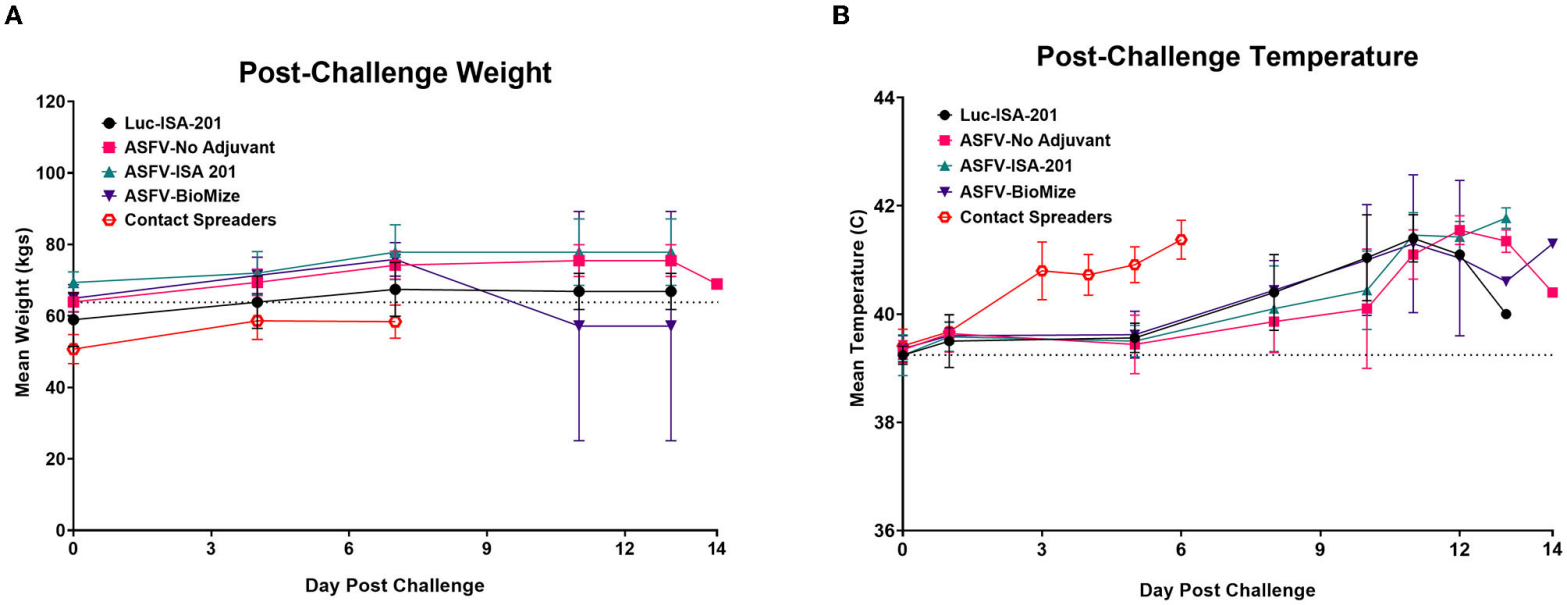
Post-challenge body temperatures and weights. Post-challenge weights **(A)** and temperatures **(B)** were collected bi-weekly and plotted as group means. Contact Spreaders were not immunized and are indicated with a red open octagon. ASFV-No Adjuvant 14 DPC weight indicates the surviving animal only. Parameters were monitored as a clinical indicator of ASFV infection and disease progression for each group.

**Figure 7:**
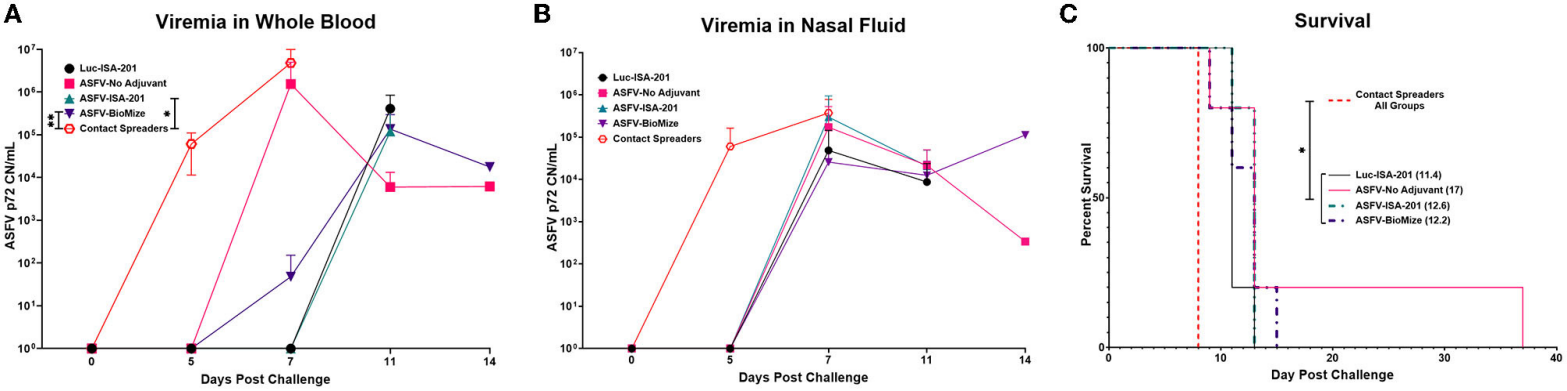
Survival and viremia post-challenge. Viremia (CN/mL) in **(A)** blood and **(B)** nasal swabs from contact non-immunized spreaders on days 0, 5, and 7; and blood from the treatment groups on days 0, 5, 7, 11, and 14 which includes the day of euthanasia for the animals that developed severe clinical disease. The mean viremia was significantly different between the contact spreaders and Montanide ISA-201™ (*p = 0.417) and BioMize^®^ (**p = 0.0439) treatment groups, any groups not denoted with an asterisk were not statistically significant and the survivor is not represented here. **(C)** Pigs were monitored and euthanized based on the severity of the disease. All the non-immunized contact spreaders (CS) are shown in red with dashed lines. Average survival is indicated in parenthesis for each group.

**Figure 8:**
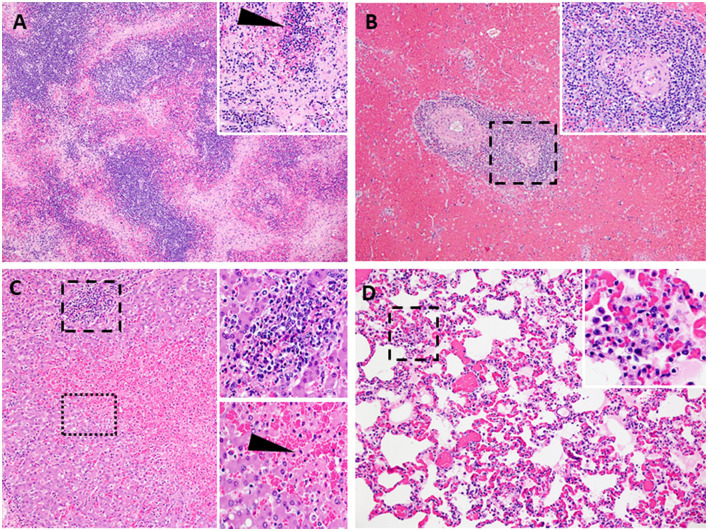
Lesions characteristic of severe acute ASF. Representative histopathology of acute ASF in pigs infected intramuscularly with ASFV strain Georgia 2007/1: Contact Spreaders 6886-8 DPC **(A–C)** and 6901-8 DPC **(D)**. **(A)** Severe lymphoid depletion, lymphocytolysis, and follicular loss accompanied by edema, fibrin, and hemorrhage (40X). (**Insert A-**100X) Vascular fibrinous degeneration (arrow), lymphocytolysis, edema, and hemorrhage (submandibular lymph node). **(B)** Marked splenic lymphoid necrosis and loss of periarteriolar sheaths with by marked diffuse red pulp hemorrhage (40X). (**Insert B-**200X) Macrophages, plasma cells, degenerate lymphocytes, eosinophils, and fibrin mats remain as periarteriolar sheaths (spleen). **(C)** Severe centrilobular necrosis of hepatocytes and marked congestion of sinusoids accompanied by moderate non-suppurative inflammation in portal regions (100X). (**Upper Insert C-**200X) Inflammation in portal regions consists of macrophages, degenerative lymphocytes, and lesser numbers of plasma cells and eosinophils. The inflammation extends beyond the portal plate into adjacent hepatic cords forming small clusters associated with hepatocyte necrosis (**Lower insert C-**200X). Junction of centrilobular hepatocyte necrosis and viable hepatocytes (liver). Necrotic hepatocytes are shrunken with hypereosinophilic cytoplasm and pyknotic nuclei, arranged in irregular cords (arrow). **(D)** Alveolar septa are congested and variably thickened by mononuclear cell infiltrates. Alveolar spaces irregularly contain aggregates of large foamy macrophages and degenerate inflammatory cells, fibrin, and edema (200X). (**Insert D-**400X) (lung).

**Figure 9:**
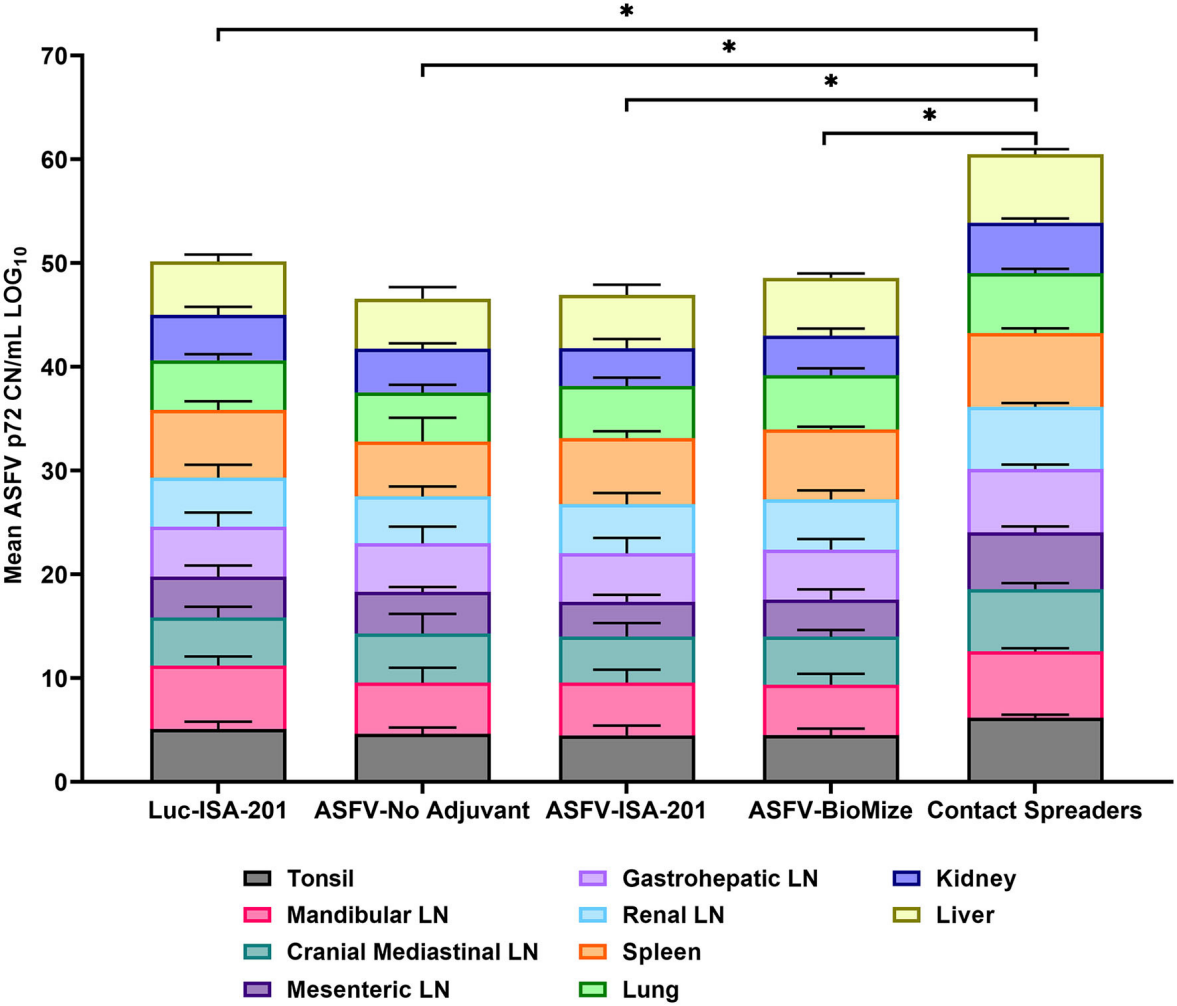
Viremia in infected tissue. Tissue CN/mL was quantified by qPCR from the samples collected on the day of euthanasia for the vaccinees and Contact Spreaders. Respective tissue types are denoted by separate colors. Each plotted value is stacked and represents the mean for each tissue. The Log base 10 was calculated for each mean CN/mL and plotted in a stacked bar graph with a total score of 100 possible and each individual tissue representative of a score between 0 to 10. Total mean ASFV p72 genomic DNA was statistically significant between the contact spreaders and all other groups denoted by asterisks, *p < 0.0001, any groups not denoted with an asterisk were not statistically significant.

**Figure 10:**
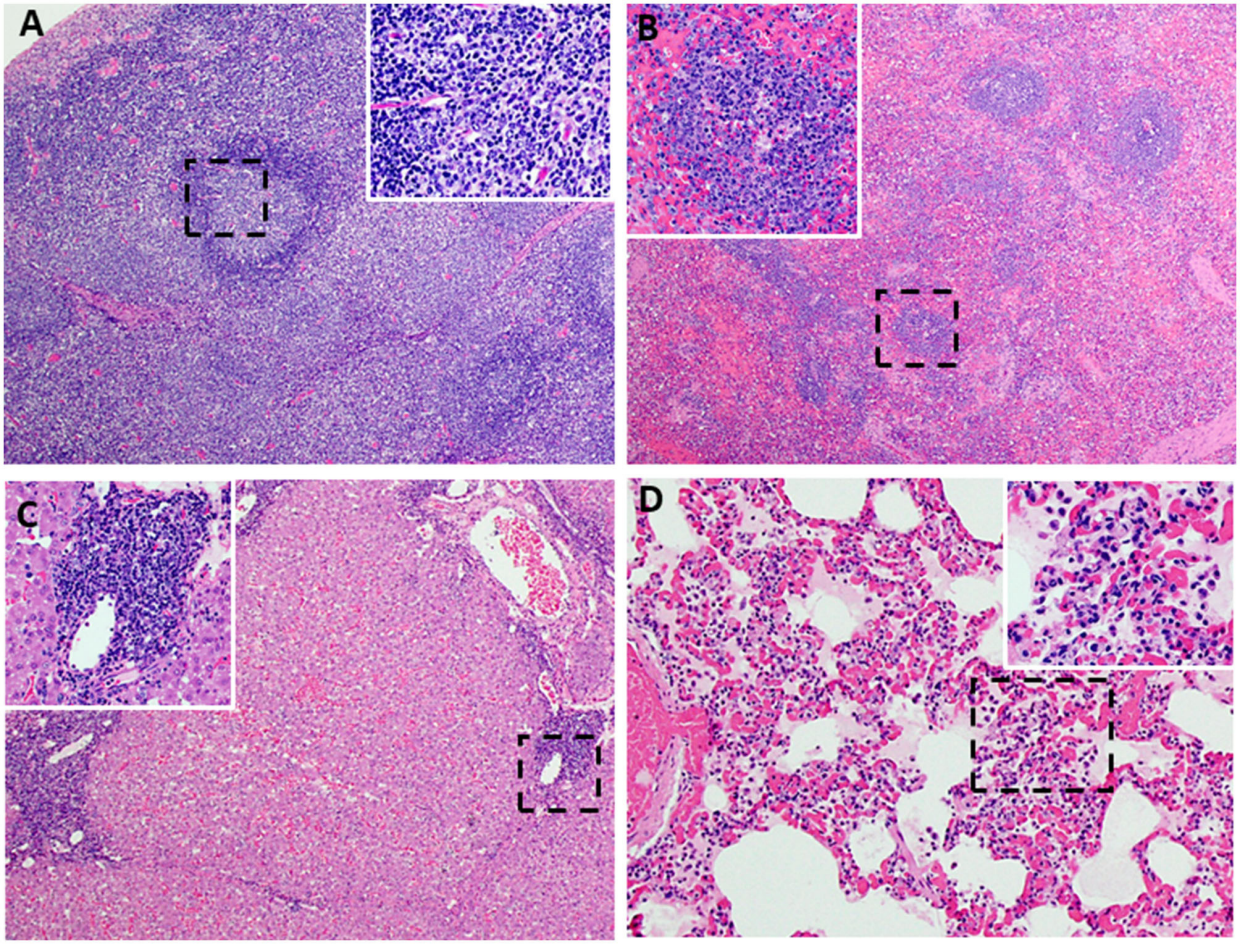
Lesions characteristic of moderate subacute ASF. Representative ASFV histopathology in pigs vaccinated with Ad5-ASFV constructs and then challenged by contact. Data from two representative pigs [6903-13 DPC: ASFV-No Adjuvant **(A–C)** and 6904-9 DPC: ASFV- BioMize **(D)**] are shown. **(A)** Minimal lymphoid depletion with variable lymphocytolysis (**Insert A-**100X) and expansion of cords and sinuses with large foamy macrophages, plasma cells, and eosinophils (40X) (submandibular lymph node). **(B)** Mild to moderate splenic lymphoid necrosis and thinning of periarteriolar sheaths accompanied by multifocal hemorrhage of the red pulp. (**Insert B**) Lymphocytolysis and necrotic cellular debris, macrophages, plasma cells, and eosinophils, (40X) (**Insert B-**200X) (spleen). **(C)** Moderate to severe, non-suppurative periportal hepatitis. Inflammation in portal regions consists of macrophages, degenerative lymphocytes, plasma cells, and lesser numbers of eosinophils, 100X (**Insert C-**200X). **(D)** Alveolar septa are markedly congested and variably thickened by mononuclear cell infiltration and cellular debris. Alveolar spaces multifocally contain aggregates of large foamy macrophages and degenerate inflammatory cells, fibrin, and edema 200X (**Insert D-**400X) (lung).

Following challenge by comingling with naïve contact spreaders, which had received IM injection of the ASFV (Georgia 2007/1), the mean pig weights in all the groups were stable for 1 week (Figure 6A). As expected, the contact spreaders developed a high fever, rapid onset of high viremia in blood as well as nasal fluids, and severe clinical disease by the time they were terminated on day 8 post-challenge (Figures 6B, 7). Histopathology of two representative contact spreaders showed tissue lesions that were consistent with severe acute ASF (Figure 8). Multiple organ and tissue samples had high virus loads, which was consistent with acute ASF observed in naïve pigs, and these values were statistically significant (*p* < 0.0001) compared with all other groups (Figure 9). Additionally, viral loads for multiple individual tissues were also found to be statistically significant when compared to the contact spreaders (see Supplementary Figures 1, 2). One week after the initiation of contact challenge, the pigs immunized with the Ad5-ASFV cocktail formulated in BioMize^®^ adjuvant had rapid weight loss that was accompanied by elevated rectal temperature, viremia in blood, and nasal fluid, and they were terminated after developing severe clinical disease (Figures 6, 7). The ASFV-BioMize immunized pigs had significantly lesser (*p* = 0.0439) viremia in their blood as compared to the contact spreaders (Figure 7A). Histopathology of one representative pig from this group revealed lesions that were consistent with moderate subacute ASF (Figure 10), but all the tissue samples collected at termination had high levels of ASFV (Figure 9). The mean weight of the negative control pigs immunized with the Ad5-Luciferase Montanide ISA-201™ formulation and the pigs immunized with the Ad5-ASFV cocktail formulated in Montanide ISA-201™ remained unchanged post-challenge, but the pigs developed a high fever in the 2^nd^ week and severe clinical disease as well as high viremia in blood and nasal fluids that necessitated termination (Figures 6, 7). Viremia in blood was found to be significantly (*p* = 0.0417) lower for the pigs that received the Ad5-ASFV cocktail formulated in Montanide ISA-201™ compared with contact spreaders, whereas the negative control pigs did not have significant levels of blood viremia compared with any other treatment groups (Figure 7A). High levels of ASFV were also detected in multiple organs and tissues (Figure 9).

Although the mean weight of the pigs immunized with the Ad5-ASFV cocktail without adjuvant was stable for 13 days, it declined thereafter as the pigs developed a high fever and rapid onset of viremia in blood and nasal fluids that peaked on day 7 after the initiation of contact challenge (Figures 7A, B). Four of the five vaccinees developed a clinical disease that necessitated termination by day 14 and histopathology of one representative pig revealed lesions that were consistent with moderate subacute ASF (Figures 7C, 10). However, tissue samples collected from the four pigs at termination had high levels of ASFV that mirrored outcomes from the other treatment groups (Figure 9). It was noted that, compared with the other groups, pigs from this group had the lowest mean viremia in the spleen (Figure 9). It was also noted that the pigs immunized with the Ad5-ASFV formulated with or without adjuvant had the lowest mean viremia in mandibular lymph nodes (Figure 9). Overall, there was no significant difference in total tissue viral load in the pigs immunized with the Ad5-ASFV formulated with or without adjuvant, when compared to the Ad5-Luciferase-negative controls (Figure 9). As expected, contact spreaders total tissue viremia was significantly higher (*p* < 0.0001) when compared with the pigs immunized with the Ad5-ASFV or the Ad5-Luciferase-negative controls (Figure 9).

### 3.5. Survivor had chronic ASF

**Figure 11:**
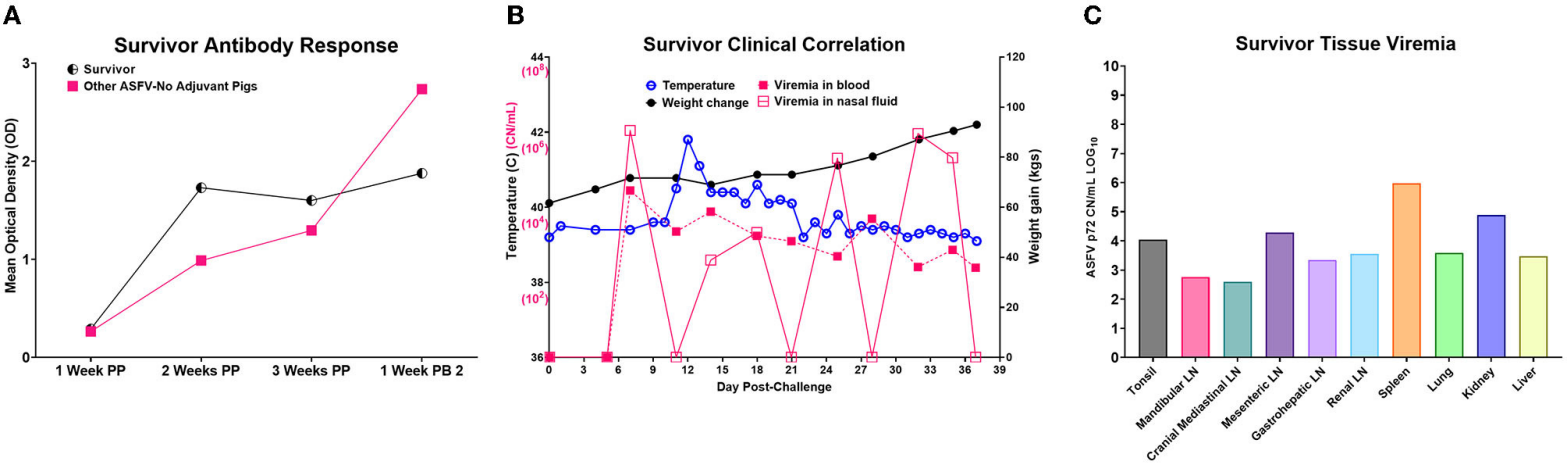
Ad5-ASFV-vaccinee that survived. **(A)** Antibody response by the lone survivor that was immunized with the Ad5-ASFV without adjuvant. The half-circle indicates the p62-specific IgG responses by the survivor compared to the mean IgG responses (solid square) by the other four group mates that succumbed after challenge. Post-prime is indicated by PP and post-boost by PB. **(B)** Temperature, weight change, and viremia in blood and nasal fluid post-challenge. **(C)** Viremia expressed in Log base 10 of the CN/mL for each respective tissue.

**Figure 12:**
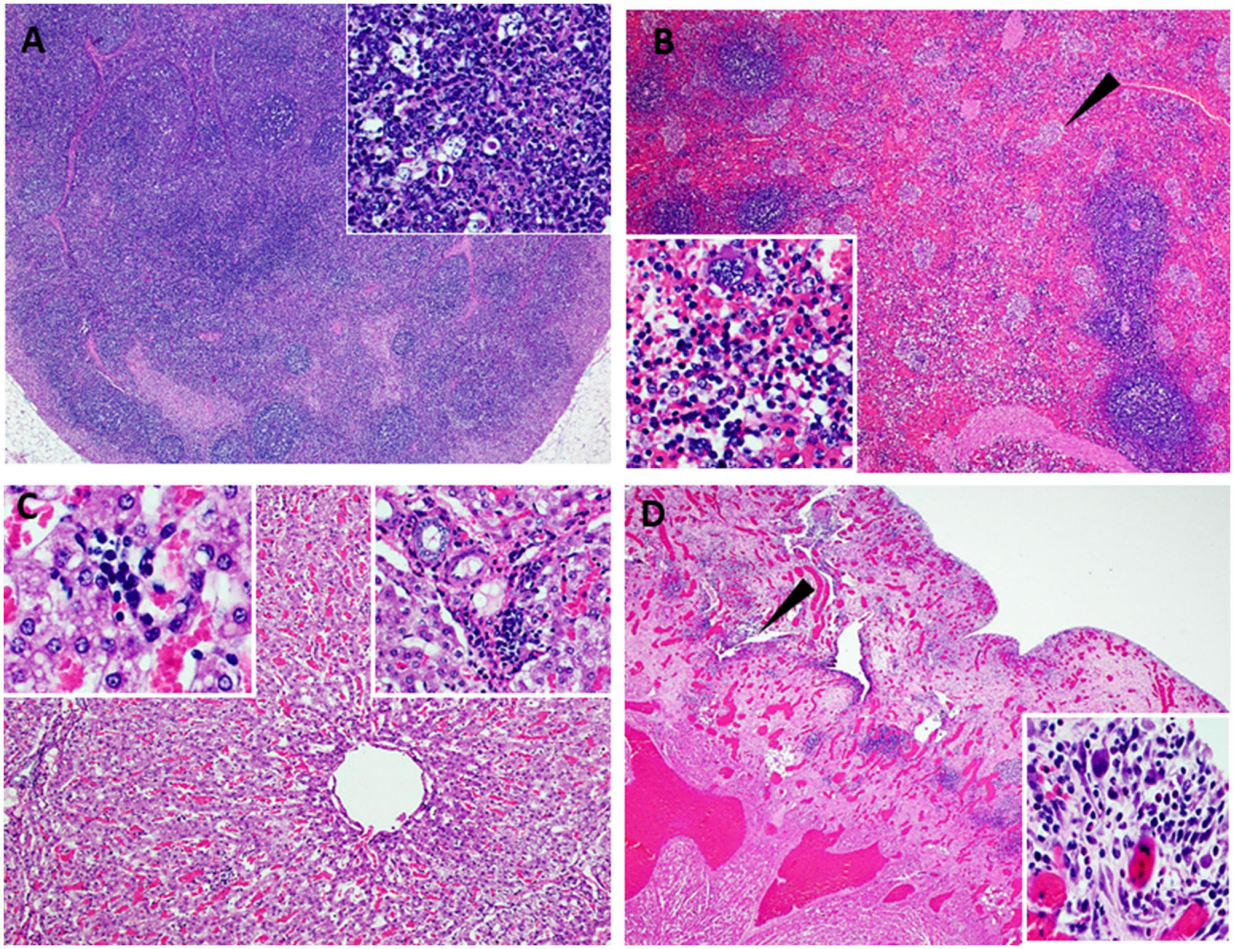
Lesions observed in chronic ASF. Atypical ASF histopathology in the survivor pig (6892-37 DPC) vaccinated with ASFV-No Adjuvant and then challenged by contact. **(A)** Moderate lymphoid hyperplasia with the expansion of follicles and cords. Variable yet infrequent lymphocytolysis occurs within germinal centers (40X) (**Insert A-**100X) (lymph node). **(B)** Moderate splenic lymphoid hyperplasia with the expansion of periarteriolar sheaths accompanied by hyperplasia of the reticular cells forming nodular bundles dispersed within the red pulp (arrow) and expansion of the red pulp with myeloid cell clusters (40X) (**Insert B-**200X) (spleen). **(C)** Moderate, non-suppurative hepatitis with moderate hepatocyte atrophy. Marked thinned hepatic cords are comprised of large irregularly arranged atrophied hepatocytes with foamy to vacuolated cytoplasm. Variable fibrosis occurs among cords and widens portal plates while multifocal, small aggregates of cellular debris, phagocytic cells, lymphocytes, and plasma cells occur among hepatic cords and in portal regions (100X) (**left and right Insert B-**200X, respectively) (liver). **(D)** Left ventricular myocardium (lower left) overlaid by markedly thickened and inflamed epicardium. Dense highly vascular fibrotic granulation tissue extends from the myocardium to the outer surface and contains numerous irregular clefts and cavitations with mixed inflammatory cells within these spaces and scattered among the edematous to fibrotic stroma (arrow). The outermost surface of the granulation tissue consists of a delicate network of capillaries, reactive fibroblasts, and numerous, often multinucleate, large histiocytic-like cells admixed with lymphocytes and plasma cells and variable numbers of eosinophils [12.5X_ (**Insert D-**200X)] (heart).

One of the five pigs (No. 6892) immunized with the Ad5-ASFV cocktail without adjuvant seroconverted and had a rapid increase in antibody response within the first 2 weeks, but unlike the other four vaccinees in this group, boosting did not significantly amplify pp62-specific IgG responses (Figure 11A). Interestingly, this pig had the lowest post-boost pp62-specific IgG responses compared with the other vaccinees in this group (Figure 11A). Following exposure to contact spreaders, viremia in blood and nasal fluid peaked on day 7 in this pig, whereas fever peaked on the 11 day (Figure 11B). The weight of the pig declined subtly from day 7, but on day 14, when the other four vaccinees in this group were terminated due to severe clinical disease, the pig started to gain weight and sustained weight gain until the study was terminated at 39 days after initiation of contact challenge (Figures 7C, 11B). The weight gain was accompanied by the resolution of fever and gradual decrease in viremia in blood until study termination (Figure 11B). Although the pig was clinically healthy at termination, it had recurrent episodes of high viremia in nasal fluid suggesting that shedding of virus and reinfection was likely still occurring (Figure 11B). Necropsy revealed that the pig had evident inflammation and marked thickening of the epicardium with an accumulation of fibrino-hemorrhagic fluid in the pericardial sac due to chronic-active pericarditis (Figure 12). In addition, histopathology revealed lesions consistent with moderate subacute ASF, but viremia in tissue samples collected at termination had much lower levels of ASFV compared with the average tissue viremia detected in the contact spreaders as well as the other treatment and control pigs (Figures 9, 11C, Supplementary Table 3). The viremia in tissue samples for the survivor was significantly lower than the contact spreaders in the mandibular LN (*p* = 0.0012), cranial mediastinal LN (*p* = 0.0029), gastrohepatic LN (*p* = 0.0344), and liver (*p* = 0.0088) (Figures 9, 11C). Significantly lower viremia in the mandibular LN of the survivor was also found compared with the pigs that received the Ad5-ASFV cocktail formulated in Montanide ISA-201™ (*p* = 0.005) and when compared to the four pigs that were also immunized without adjuvant (*p* = 0.0421) who succumbed earlier in the study (Figures 9, 11C).

### 3.6. Clinical disease and histopathology at termination

Following initiation of challenge by contact with naïve ASFV-infected pigs, which were terminated on day 8, the mean survival of the negative control pigs (Ad5-Luciferase Montanide ISA-201™ formulation) was 11.4 days compared with 12.2 to 17 days for Ad5-ASFV vaccinees (Ad5-ASFV cocktail without adjuvant, Ad5-ASFV cocktail Montanide ISA-201™ formulation, and Ad5-ASFV cocktail BioMize^®^ formulation) with the exemption of one vaccinee that survived (Ad5-ASFV cocktail without adjuvant) until study termination on day 39 (Figure 7C). Typical symptoms for all animals that succumbed to the disease consisted of high fever, lethargy, reddening of the skin, anorexia, and weight loss. Analysis of clinical presentation of ASF at termination showed that the vaccinees, which were immunized with the Ad5-ASFV cocktail formulated with Montanide ISA-201™ or BioMize^®^ had an average clinical score of 3.2, while the vaccinees that were immunized with the Ad5-ASFV cocktail without adjuvant had an average clinical score of 3.4 (Supplementary Table 4). Although these scores were less than the average clinical score of 3.6 for the naïve contact spreaders, the difference was not significant (Supplementary Table 4). The negative controls (Ad5-Luciferase Montanide ISA-201™ formulation) were a notable exemption with an average clinical score of 1.4 and the lone survivor (Ad5-ASFV cocktail without adjuvant) had no clinical symptoms at termination (Supplementary Table 4).

**Figure 13:**
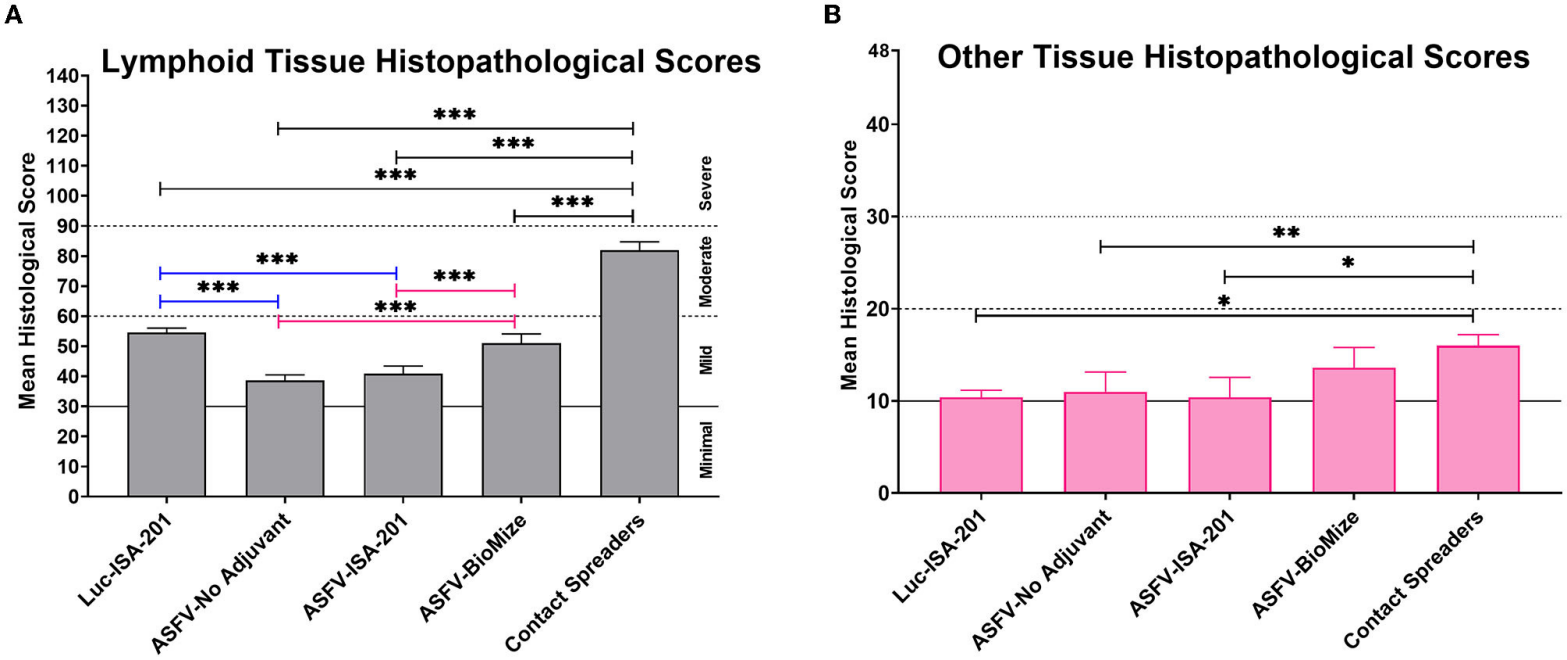
Tissue histopathology. Mean histological scores per group for **(A)** lymphoid tissue and **(B)** other tissues (liver, lungs, kidney, and heart). Group comparisons are indicated by ^*^p = 0.0014, ^**^p = 0.0057, ^***^p < 0.0001, and any groups not denoted with an asterisk were not statistically significant.

Compared with the contact spreaders, the mean histopathological scores of lymphoid tissues from the Ad5-Luciferase controls and the vaccinees immunized with the Ad5-ASFV formulated with or without adjuvant were significantly (*p* < 0.0001) lower (Figure 13A). It was also noted that the mean histopathological scores of lymphoid tissues from the pigs immunized with Ad5-ASFV without adjuvant or the Ad5-ASFV Montanide ISA-201™ vaccinees were significantly (*p* < 0.0001) lower than the mean scores for lymphoid tissues from the Ad5-Luciferase controls and the Ad5-ASFV BioMize^®^ vaccinees (Figure 13A). There was no difference between the mean scores for the lymphoid tissues from the pigs immunized with the Ad5-ASFV without adjuvant and the Ad5-ASFV Montanide ISA-201™ vaccinees (Figure 13A). The mean histopathological scores for visceral organs (liver, lungs, and kidney) from the contact spreaders were significantly (*p* = 0.0014) higher than the mean score for Ad5-Luciferase controls and the Ad5-ASFV Montanide ISA-201™ vaccinees as well as the mean score for the vaccinees that received the Ad5-ASFV without adjuvant (*p* = 0.0057) (Figure 13B). There was no difference between the contact spreaders and the Ad5-ASFV BioMize^®^ vaccinees (Figure 13B). However, analyses of individual lymphoid tissues as well as visceral organs showed that there was extreme variation in histopathological scores between the groups and among the controls as well as the vaccinees (Supplementary Figures 3, 4).

## 4. Discussion

Promising results have been obtained from recent studies that evaluated the protective efficacy of ASFV prototype vaccines, primarily attenuated virus (8, 9, 11–13). Despite these advancements, the need for safe and more efficacious ASFV vaccines has been made increasingly evident given the poor performance of the vaccine candidates, the threat posed by the continued virus global spread, and significant economic losses associated with this disease (14, 41). This study evaluated the safety, tolerability, and efficacy of experimental subunit vaccines that were formulated, with and without adjuvant, using a cocktail of 42 replication-deficient adenovirus-vectored multicistronic expression cassettes encoding ASFV antigens. As protective ASFV antigens are yet to be identified, immunization with a cocktail of the multicistronic expression cassettes was a strategy to deliver nearly 100% of the ASFV Georgia 2007/1 proteome to mimic antigen delivery by some ASFV mutants that have been shown to confer protection and if successful, this approach could fast track identification of the protective proteins needed for the development of rationally designed prototype subunit vaccines (8–13, 42). The multicistronic expression cassettes were stable after multiple passages of the recombinant adenoviruses as judged by expression of the FLAG tag at the C-terminal of the last gene in the cassette. More importantly, the proteins expressed by the recombinant adenoviruses were shown to be authentic ASFV antigens as judged by flow cytometric analyses using ASFV convalescent serum (Figure 1, Supplementary Table 1). However, *in vitro* expression of the ASFV antigens by the recombinant adenoviruses was heterogeneous ranging from low (e.g., Ad5-05, Ad5-13), medium (e.g., Ad5-18, Ad5-39), to high (e.g., Ad5-09, Ad5-19) expression (Figure 1, Supplementary Table 1). This outcome could have been influenced by the gene combination or arrangement in each cassette, but it was noted that protein expression by the majority of the cassettes was comparable or better than the positive control recombinant adenovirus encoding the pp62 antigen alone (Figure 1, Supplementary Table 1). It is possible that the heterogeneous expression of the ASFV antigens could have had an impact on the magnitude of priming B cell and T cell responses, but this study did not ascertain whether this was reflected in the resultant immune responses.

Three doses of a cocktail of the recombinant adenoviruses expressing the ASFV antigens (Ad5-ASFV), formulated with or without adjuvant, were well tolerated as all the pigs in the treatment groups remained healthy throughout the immunization phase (Figure 3). The Ad5Luciferase-negative control construct formulated with the Montanide ISA-201™ adjuvant (Luc-ISA-201) was also well tolerated (Figure 3). Notably, the priming dose of the Ad5-ASFV cocktail alone or formulated with the Montanide ISA-201™ adjuvant (ASFV-ISA-201), but not the Ad5-ASFV cocktail formulated in BioMize^®^ adjuvant (ASFV-BioMize), elicited significant (*p* < 0.0001) pp62-specific IgG responses compared with the Ad5-Luciferase-negative control immunogen (Figure 4B). Boosting significantly (*p* < 0.0001) recalled pp62-specific IgG responses in the vaccinees, but there was no difference in pp62-specific IgG responses between the pigs immunized with the Ad5-ASFV cocktail alone and the pigs immunized with either the Ad5-ASFV ISA-201 or the Ad5-ASFV BioMize^®^ formulations, suggesting that the adjuvants were not necessary (Figure 4C). This outcome cannot be used to imply that the adjuvants did not have an effect on the antibody isotype or T cell responses and/or functions of the induced effectors as these were not evaluated in this study. More importantly, the induced antibodies strongly recognized ASFV-infected, but not uninfected, primary swine cells (Figure 5). This outcome confirmed that the Ad5-ASFV immunogens generated using synthetic genes induced authentic IgG responses, which was consistent with previous findings in domestic pigs and wild boars (7, 21, 22, 37, 38, 43, 44).

Although homologous prime-boost immunization of pigs induced and significantly (*p* < 0.0001) expanded antibody responses as judged by tracking pp62-specific IgGs, all the controls and the vaccinees, with one exemption, succumbed to ASF within 14 days following exposure to comingled naïve contact spreaders that had received IM injection of ASFV (Georgia 2007/1) (Figures 6, 7). The challenge by contact with infected pigs, as opposed to the needle challenge, is the ideal model for natural infection given that transmission of ASFV by ticks is limited to Sub-Saharan Africa, while in other regions of the world, the spread of virus to naïve pigs is by ingestion of contaminated materials or exposure to infected pigs (36). In this natural ASFV transmission model, the infection may occur via the mucosal route through direct animal-to-animal contact or oral–fecal route, likely with multiple low-dose repeated exposures more representative of non-tick ASFV transmission (36). This challenge model has previously been shown to be effective by other investigators using ASFV (Arm07 genotype II isolate) (7, 36). Seven days after the initiation of the challenge, the pigs immunized with the Ad5-ASFV cocktail formulated with or without adjuvant, lost weight, and developed high fever and viremia in blood as well as in nasal fluids (Figures 6, 7A, B). Blood viremia was significantly lower in the pigs immunized with the Ad5-ASFV cocktail formulated in adjuvant compared with the naïve contact spreaders (Figure 7A). The pigs were terminated by day 14 after they developed clinical ASF (Figure 7C). There was no significant difference in clinical presentation of ASF at termination between the naïve contact spreaders and the treatment groups (Supplementary Table 4). The Ad5-Luciferase controls with an average clinical score of 1.4 and the survivor (immunized with the Ad5-ASFV cocktail without adjuvant) which had no clinical symptoms at termination were the only exemptions (Supplementary Table 4). The low clinical score noted for the Ad5-Luciferase controls could have been due to the presentation of peracute ASF where animals are viremic but can show little to no outward signs of disease (45, 46).

Histopathology of representative pigs revealed lesions that were consistent with moderate subacute ASF (Figures 7C, 10). These outcomes were consistent with the observations made from the naïve spreaders as well as the Ad5-Luciferase-negative controls (Figures 6–8). At termination, the mean viremia in tissues from the naïve contact spreaders was significantly (*p* < 0.0001) higher compared with the mean viremia in tissues from the Ad5-Luciferase-negative controls and the pigs immunized with the Ad5-ASFV cocktail alone or formulated in adjuvant (Figure 9). However, there was no significant difference in tissue viral load in the Ad5-ASFV vaccinees when compared to the Ad5-Luciferase-negative controls (Figure 9). In contrast, it was observed that the mean histopathological scores of tissues from the contact spreaders were higher than the mean scores for the Ad5-Luciferase controls and the Ad5-ASFV vaccinees (Figure 13). However, at the individual level, there was wide variation in total tissue histopathological scores between the treatment groups and among the individual control and vaccinated pigs (Supplementary Figures 3, 4). Overall, the outcome showed that immunization of pigs with the experimental Ad5-ASFV formulation was not protective even though the immunogens induced antibody responses that recognized ASFV-infected cells. However, only anti-pp62 antibody responses were evaluated, and therefore, it is not known whether the vaccinees mounted antibodies against the other antigens included in the cocktail. Although ASFV neutralization has been reported, the role of antibodies in protection against ASFV infection is yet to be determined, but it might depend on the target antigens or subtype of immunoglobulin induced (47–50).

One pig (No. 6892) immunized with the Ad5-ASFV cocktail without adjuvant was the only survivor (Figure 7C). Although the pig mounted a rapid increase in pp62-specific IgG responses after priming, there was no significant increase in antibody response after boosting and it had the lowest antibody responses compared with the other four pigs in this group (Figure 11A). It was also noted that viremia peaked 1 week after initiation of contact challenge, followed by subtle weight loss and high fever that peaked on the 12^th^ day (Figure 11B). By day 14 when all the other pigs in this group were terminated, the pig started to gain weight and sustained it for the next 25 days during which time the fever resolved and viremia in blood gradually decreased, but there were recurrent episodes of high viremia in nasal fluid until study termination (Figures 7C, 11B). The viral detection in nasal fluid likely indicates transient reinfection from the environment for this pig; however, the environmental viral loads were not monitored in this study to confirm this possibility. The pig did not exhibit typical ASF clinical symptoms, but necropsy revealed that it had chronic pericarditis and lesions consistent with moderate subacute ASF (Figures 11, 12). At termination, tissue samples had the lowest amount of ASFV compared with the average viremia detected in the contact spreaders as well as the pigs from all the other groups (Figures 9, 11C). The recurring virus shedding, low viremia in tissues, and the lesions observed were consistent with outcomes reported in pigs with chronic ASF (46, 51–56). Although this pig had the lowest anti-pp62 antibodies, B cell responses against other antigens included in the Ad5-ASFV cocktail were not determined and in addition, T cell responses were not evaluated. A previous study has shown that, following immunization, vaccinees that mounted the lowest, but not the highest, antigen-specific IgG responses had better survival rates and lower clinical scores (23). It is possible that the survivors mounted low antibody responses that were directed against protective determinants, whereas the non-survivors had strong responses against non-protective antigens. It is also likely that the sole survivor had unique genetic traits that enabled it to resolve the virus which would be consistent with the observation that infection of naïve pigs with the most virulent ASFV isolates does not always result in 100% mortality (57–60).

In conclusion, the results from this study showed that immunization of pigs with a cocktail of 42 multicistronic ASFV antigen expression constructs, formulated with and without adjuvant, primed humoral immune responses as judged by anti-pp62 IgGs, which underwent significant recall after boosting. However, none of the Ad5-ASFV formulation conferred protection upon challenge except one sole survivor that had been immunized with the Ad5-ASFV cocktail without adjuvant. Even though the survivor had no typical clinical symptoms, they had viral loads and lesions consistent with chronic ASF. Given the near 100% coverage of the ASFV proteome, the outcome from this study suggests that *in vivo* antigen expression, but not the antigen content, might be the limitation of this immunization approach. This could be due, in part, to the fact that the replication-incompetent adenovirus does not mimic the replication of attenuated ASFV as it does not amplify and persist in pigs to effectively prime and expand protective immunity. Additionally, the Ad5 vector and live attenuated ASF virus do not use identical promoters or processes for gene expression, with attenuated ASFV genes being differentially transcribed ranging from early to late expression while the Ad5-vectored multicistronic genes rely on a single CMV promoter for simultaneous co-expression. Protein expression by the Ad5-ASFV constructs was heterogeneous, and this outcome could have been influenced by the gene combination or sequence in each cassette. The heterogeneous antigen expression could have had an impact on the magnitude of priming B cell and T cell responses, but this study did not ascertain whether this was reflected in the resultant immune responses. Addressing the limitations of the *in vivo* ASFV antigen delivery will likely yield promising outcomes. Based on our previous studies, the homologous prime-boost immunization regimen using replication-incompetent Ad5 is well suited for induction and robust expansion of B cell, but not T cell responses (21, 22). Future approaches will evaluate protective efficacy of replication-competent vaccine vectors expressing the multicistronic ASFV cassettes as well as heterologous prime-boost immunization (7, 23, 24, 29, 61, 62). Other limitations of this study include the use of a small sample size (*n* = 5), but addressing this limitation is still a challenge as ABSL-3Ag biocontainment space required for ASFV challenge has limited capacity.

## Data availability statement

The original contributions presented in the study are included in the article/Supplementary material, further inquiries can be directed to the corresponding authors.

## Ethics statement

The animal study was reviewed and approved by Kansas State University Institutional Animal Care and Use Committee (IACUC).

## Author contributions

WM: conceptualization and supervision. NS, SL, MZ, JY, RK, KM, JM, LB, HS, EH, TB, TK, KA, and JT: methodology. KA and JT: pathology. MZ, JT, and WM: data curation and writing, review, and editing. WM and SW: funding acquisition. All authors have read and agreed to the published version of the manuscript.
