# Supplementary material for: Immunization of pigs with replication-incompetent adenovirus-vectored African swine fever virus multi-antigens induced humoral immune responses but no protection following contact challenge
Source: Front Vet Sci. 2023 Jun 19;10:1208275. doi: 10.3389/fvets.2023.1208275 (PMC10316028; doi:10.3389/fvets.2023.1208275)
Supplement: Supplementary file 1 [file Data_Sheet_1.pdf]

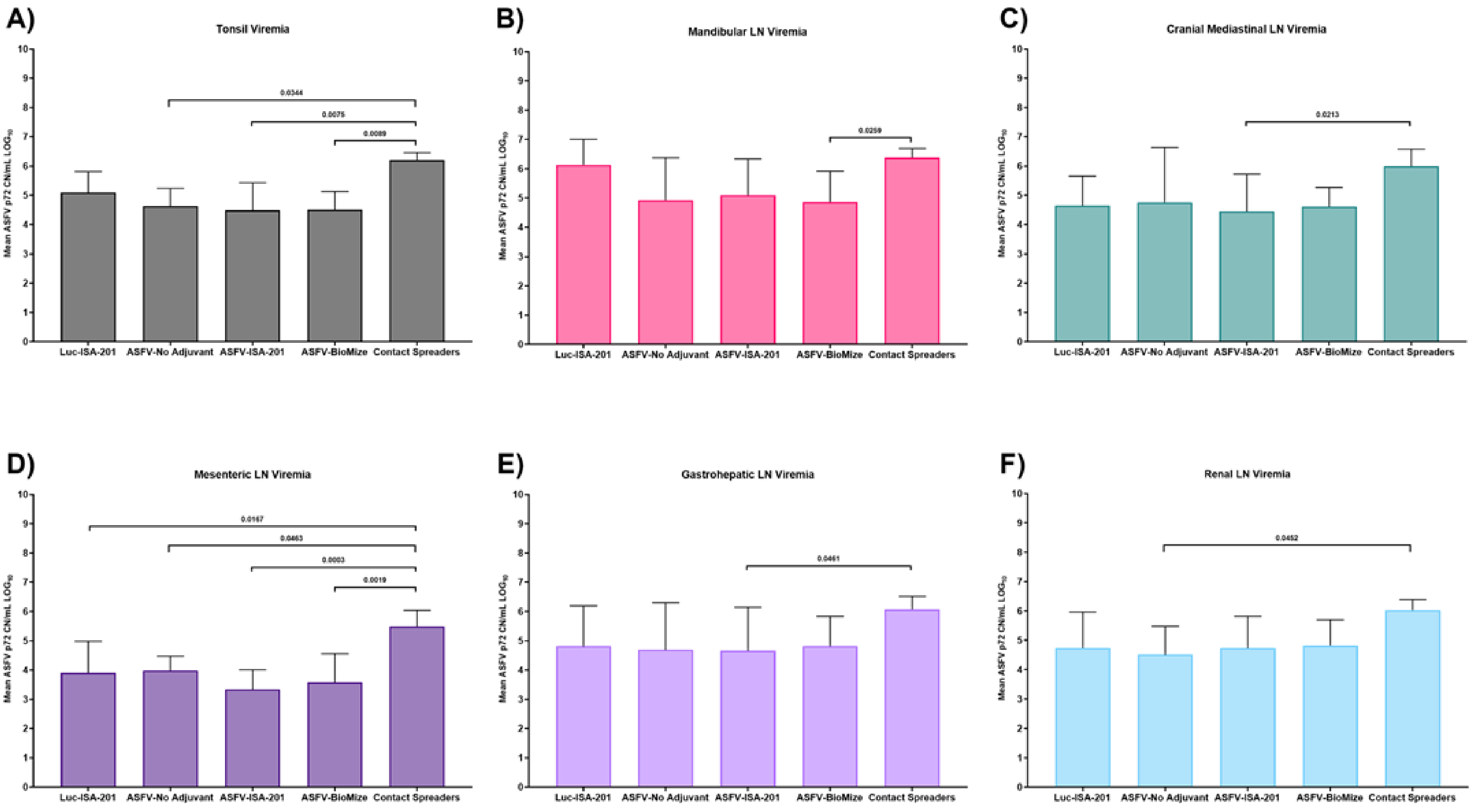

Supplementary Figure 1. Viremia in infected lymphoid tissue. Tissue CN/mL was quantified by qPCR from the samples collected on the day of euthanasia for the vaccinees and Contact Spreaders for (**A**) tonsil, (**B**) mandibular LN, (**C**) cranial mediastinal LN, (**D**) mesenteric LN, (**E**) gastrohepatic LN, and (**F**) the renal LN. Each plotted value represents the mean for each tissue per group. The Log base 10 was calculated for each mean CN/mL and plotted as supplementary to the Figure 9 stacked bar graph. Total mean ASFV p72 genomic DNA was statistically significant between the groups as denoted by the p-value indicated on each graph, any groups not denoted were not statistically significant.

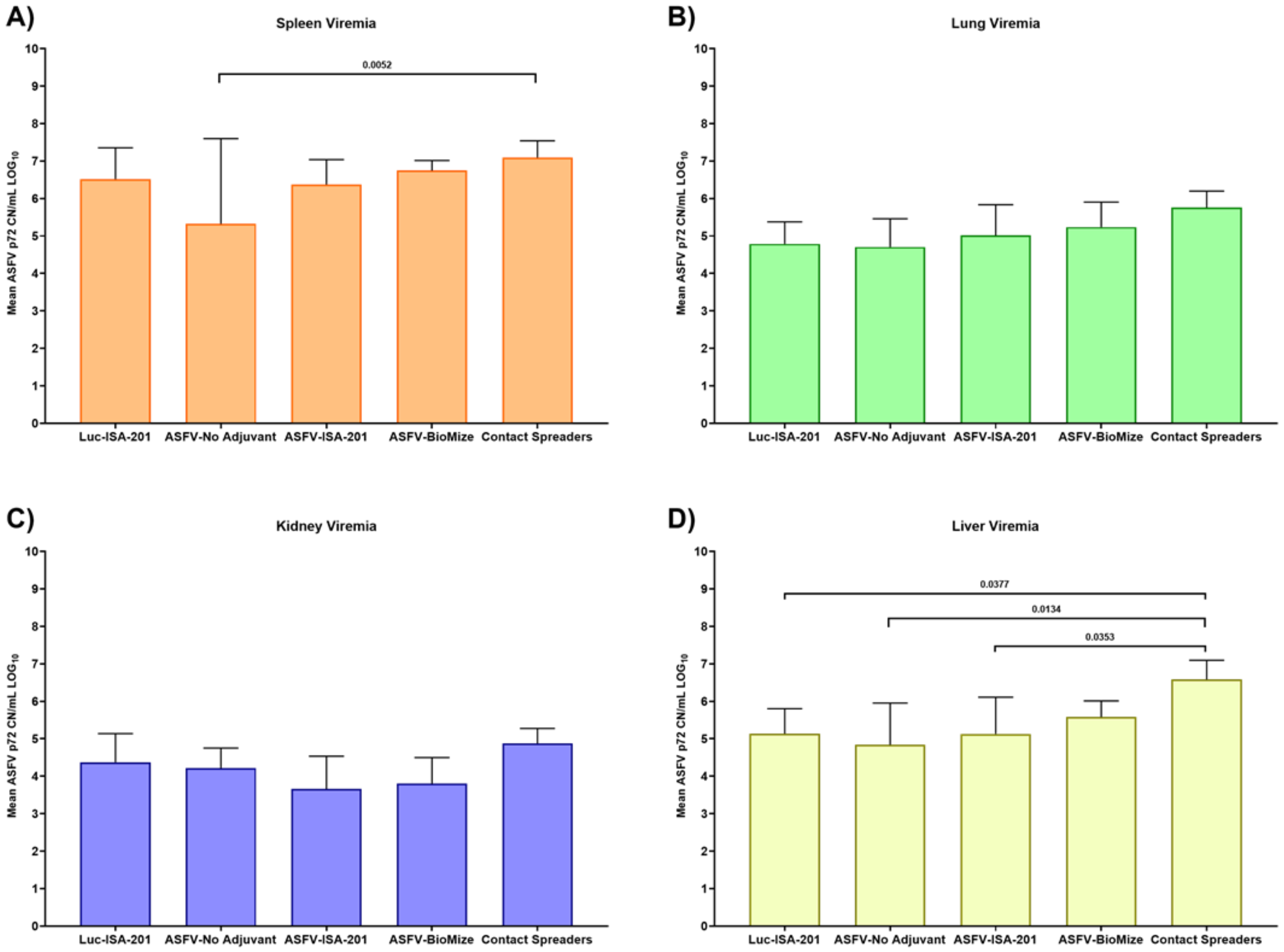

Supplementary Figure 2. Viremia in infected visceral tissue. Tissue CN/mL was quantified by qPCR from the samples collected on the day of euthanasia for the vaccinees and Contact Spreaders for (**A**) the spleen, (**B**) the lung, (**C**) the kidney, and (**D**) the liver. Each plotted value represents the mean for each tissue per group. The Log base 10 was calculated for each mean CN/mL and plotted as supplementary to the Figure 9 stacked bar graph. Total mean ASFV p72 genomic DNA was statistically significant between the groups as denoted by the p-value indicated in (**A**) and (**D**), any groups not denoted were not statistically significant.

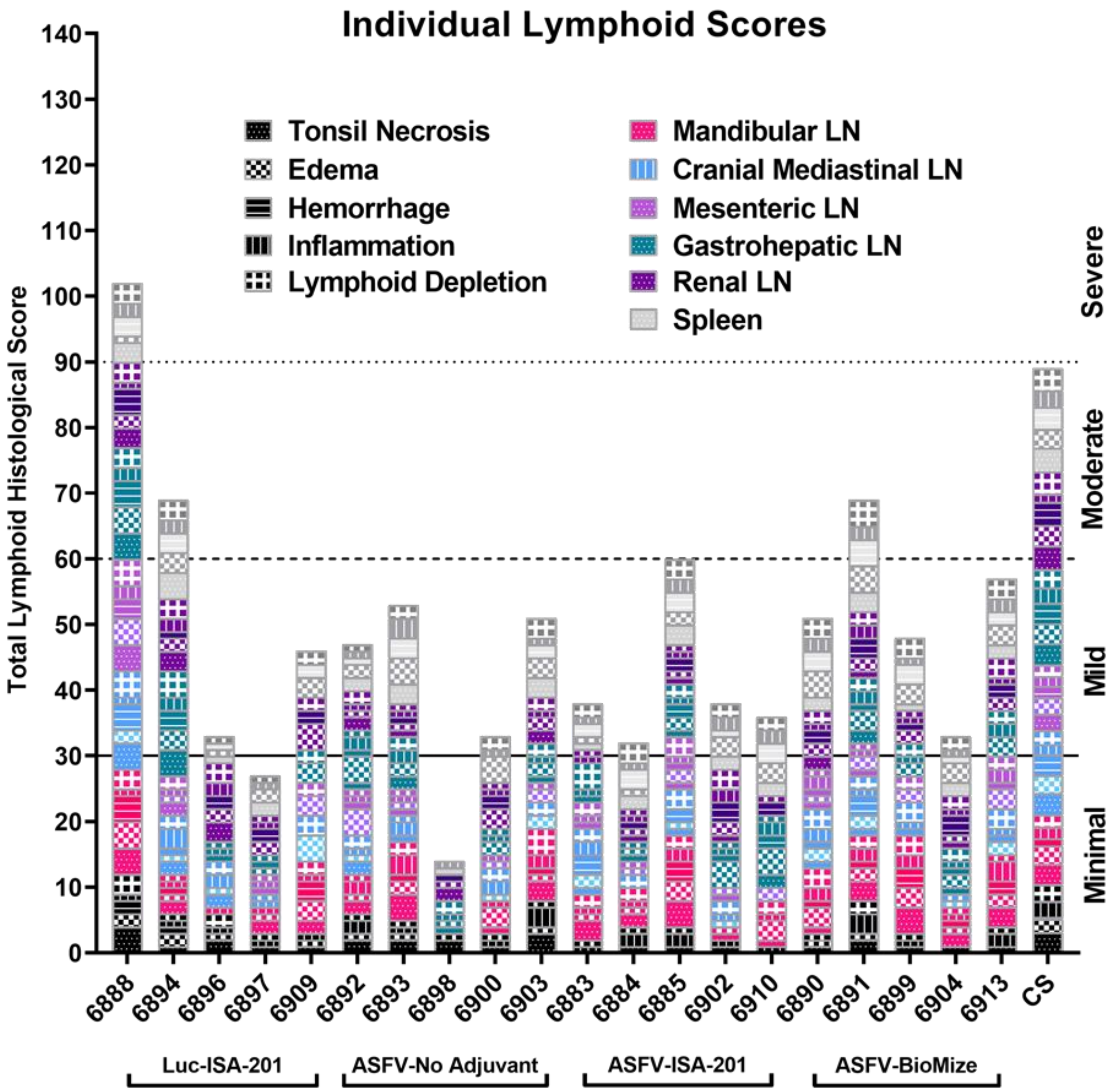

Supplementary Figure 3. Lymphoid tissue histopathology. Individual lymphoid tissue histological scores for each pig in the treatment and control groups, whereas the Contact Spreaders (CS) are plotted as group mean. Histological scores are stacked and reflected as the overall total per animal.

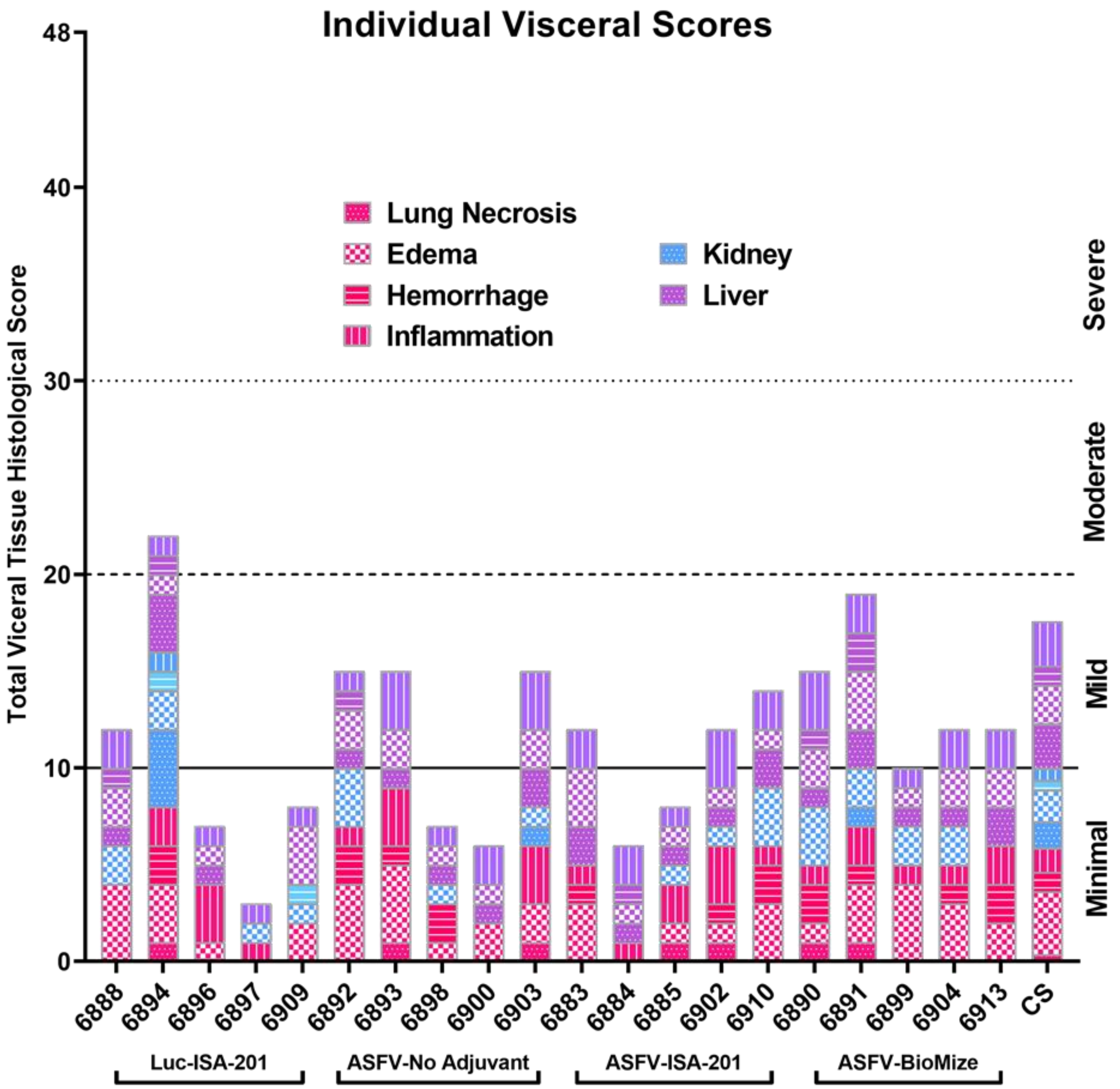

Supplementary Figure 4. Visceral tissue histopathology. Individual visceral tissue histological scores for each pig in the treatment and control groups, whereas the Contact Spreaders (CS) are plotted as group mean. Histological scores are stacked and reflected as the overall total per animal.

Supplementary Table 1. ASFV Antigen expression.

	Construct	% Positive cells
	**None (negative control)**	0.07
	**Ad5-pp62 (positive control)**	2.97
**Low ASFV antigen Expression**	**Ad5-05**	0.53
	**Ad5-13**	0.75
	**Ad5-34**	1.53
	**Ad5-03**	1.71
	**Ad5-01**	1.92
**Medium ASFV antigen Expression**	**Ad5-04**	2.38
	**Ad5-29**	2.87
	**Ad5-11**	3.08
	**Ad5-10**	3.14
	**Ad5-18**	3.29
	**Ad5-39**	3.44
**High ASFV antigen Expression**	**Ad5-21**	4.55
	**Ad5-09**	7.78
	**Ad5-19**	8.92

Values are expressed as a percent of the total parental cell population as measured using ASFV convalescent serum by the Ad5-ASFV constructs using flow cytometric analyses.

Supplementary Table 2. Clinical Score Rubric.

Parameter	Score	Description
Breathing	0	Normal breathing, no coughing/sneezing
	1	Slightly labored breathing, mild coughing/sneezing
	2	Labored, increased rate, moderate coughing/sneezing
	3	Highly labored, rapid rate, frequent coughing/sneezing
Liveliness	0	Attentive (curious, stands up immediately)
	1	Slightly reduced (stands up hesitantly but without help)
	2	Tired, get up only when forced to, lies down again
	3	Dormant, will not stand up
Walking	0	Well-coordinated movements
	1	Stiffness while standing up, afterward normal
	2	Distinct lameness, able to walk
	3	Massive lameness, unable to walk
Skin	0	Evenly light pink, flat hair coat
	1	Reddened skin areas
	2	Purple/blue discolored areas (ears, legs)
	3	Black-red discoloration of the skin
Eyes/conjunctiva	0	Light pink
	1	Reddened, clear secretion
	2	Highly inflamed, turbid secretion
	3	Highly inflamed, secretions prevent eyes from opening
Body Condition [detection of ribs, back bones, hip bones, and pin bones]	0	Ideal, smooth, and rounded with an even feel
	1	Mildly Thin, easy to feel with pressure
	2	Moderately Thin, easy to feel with pressure, and beginning to see
	3	Emaciated, obvious/easy to see
Feces	0	normal
	1	None
	2	Diarrhea
	3	Mucus or bloody diarrhea

Maximum score: 21

Supplementary Table 3. Average ASFV tissue viremia per group compared to surviving pig (CN/mL).

Groups	Tonsil	Mandibular LN	Cranial Mediastinal LN	Mesenteric LN	Gastrohepatic LN	Renal LN	Spleen	Lung	Kidney	Liver
Survivor	3.9E+02	2.2E+03	7.7E+04	2.9E+03	3.9E+03	5.7E+02	2.0E+04	3.6E+03	9.5E+05	1.1E+04
Luc-ISA-201	3.3E+05	5.9E+06	2.0E+05	4.9E+04	5.2E+05	2.7E+05	1.1E+07	1.2E+05	4.6E+04	3.4E+05
ASFV-No Adjuvant	9.1E+04	3.6E+05	1.2E+07	1.5E+04	1.4E+06	1.2E+05	9.6E+06	9.4E+04	2.7E+04	3.9E+05
ASFV-ISA-201	1.6E+05	6.5E+05	1.6E+05	5.4E+03	5.8E+05	2.3E+05	5.9E+06	3.1E+05	1.4E+04	5.7E+05
ASFV-BioMize	6.4E+04	4.1E+05	8.9E+04	3.3E+04	3.7E+05	4.4E+05	6.5E+06	4.9E+05	2.1E+04	5.8E+05
Contact Spreaders	1.9E+06	3.0E+06	1.8E+06	6.3E+05	1.8E+06	1.4E+06	1.7E+07	1.1E+06	1.1E+05	7.9E+06

Supplementary Table 4. Terminal clinical scores.

Groups	Pig ID	Adjuvant	Terminal Clinical Score	Group Mean Clinical Score
Contact Spreaders	6908	none	1	3.6
	6886		6	
	6915		4	
	6901		4	
	6887		4	
	6906		4	
	6912		3	
	6907		3	
Luc-ISA-201	6894	Montanide ISA-201™	1	1.4
	6909		2	
	6896		1	
	6897		2	
	6888		1	
ASFV-No Adjuvant	6898	No Adjuvant	9	3.4
	6903		2	
	6893		3	
	6892		0	
	6900		3	
ASFV-ISA-201	6884	Montanide ISA-201™	9	3.2
	6885		1	
	6883		1	
	6902		2	
	6910		3	
ASFV-BioMize	6913	BioMize®	5	3.2
	6891		1	
	6890		2	
	6899		2	
	6904		6	
